# Extracellular matrix remodeling in tumor progression and immune escape: from mechanisms to treatments

**DOI:** 10.1186/s12943-023-01744-8

**Published:** 2023-03-11

**Authors:** Zhennan Yuan, Yingpu Li, Sifan Zhang, Xueying Wang, He Dou, Xi Yu, Zhiren Zhang, Shanshan Yang, Min Xiao

**Affiliations:** 1 Department of Oncological Surgery, Harbin Medical University Cancer Hospital, Harbin, 150081 China; 2 Department of Neurobiology, Harbin Medical University, Harbin, 150081 China; 3 Department of Otolaryngology Head and Neck Surgery, Xiangya Hospital, Central South University, Changsha, 410008 China; 4 Department of Gynecological Oncology, Harbin Medical University Cancer Hospital, Harbin, 150081 China; 5 NHC Key Laboratory of Cell Transplantation, The First Affiliated Hospital of Harbin Medical University, Harbin, 150001 China; 6 Institute of Metabolic Disease, Heilongjiang Academy of Medical Science, Heilongjiang Key Laboratory for Metabolic Disorder and Cancer Related Cardiovascular Diseases, Harbin, 150001 China; 7 Department of Gynecological Radiotherapy, Harbin Medical University Cancer Hospital, Harbin, 150000 China

**Keywords:** Extracellular matrix, Cancer metabolism, Immune escape, Collagen orientation, Stiffness, Immunotherapy

## Abstract

The malignant tumor is a multi-etiological, systemic and complex disease characterized by uncontrolled cell proliferation and distant metastasis. Anticancer treatments including adjuvant therapies and targeted therapies are effective in eliminating cancer cells but in a limited number of patients. Increasing evidence suggests that the extracellular matrix (ECM) plays an important role in tumor development through changes in macromolecule components, degradation enzymes and stiffness. These variations are under the control of cellular components in tumor tissue via the aberrant activation of signaling pathways, the interaction of the ECM components to multiple surface receptors, and mechanical impact. Additionally, the ECM shaped by cancer regulates immune cells which results in an immune suppressive microenvironment and hinders the efficacy of immunotherapies. Thus, the ECM acts as a barrier to protect cancer from treatments and supports tumor progression. Nevertheless, the profound regulatory network of the ECM remodeling hampers the design of individualized antitumor treatment. Here, we elaborate on the composition of the malignant ECM, and discuss the specific mechanisms of the ECM remodeling. Precisely, we highlight the impact of the ECM remodeling on tumor development, including proliferation, anoikis, metastasis, angiogenesis, lymphangiogenesis, and immune escape. Finally, we emphasize ECM "normalization" as a potential strategy for anti-malignant treatment.

## Background

Tumor cells and the tumor microenvironment (TME) constitute the main part of solid tumors. TME is composed of multiple cellular components, including fibroblasts, endothelial cells (ECs), immunocytes, adipocytes, and acellular component: the extracellular matrix (ECM) [1]. The occurrence and the development of malignant tumors depend on the extracellular signals and is not a completely autonomous process of tumor cells [2, 3]. Besides of intercellular contact, signaling transductions are mostly dependent on acellular components, which contains not only bioactive agents but also mechano-transductal properties. As an acellular component in the TME, the ECM attracts more attention from scientists as a key factor in cancer progression.

The ECM mainly consists of proteoglycans, glycoproteins, matricellular proteins (including secreted proteins acidic and rich in cysteine (SPARC/osteonectin), osteopontin (OPN/SPP-1), thrombospondin (THBS/TSP)) and structural proteins such as tenascin, collagen and laminin [4–6]. The ECM remodeling, which featured by the changes on the content, activity and crosslinking of these proteins trigger the variations of signal transduction. In most tumor tissues, the ECM remodeling is characterized by increased collagen synthesis and deposition, usually accompanied by the expression of remodeling enzymes such as matrix metalloproteinases (MMPs), lysyl oxidase (LOX), lysyl oxidase-like proteins (LOXLs), WNT1-inducible signaling pathway proteins (WISPs) and others [7]. These enzymes can treat specific ECM components as substrates and catalyze them to control tissues stiffness and cell–matrix interactions through their unique biochemical and physical properties [8]. Some enzymes process matrix components, such as collagen, resulting in the production and release of bioactive fragments [9, 10]. Amount of changes in the expression level of MMPs in the tumor microenvironment represent the malignant degree of the tumor, reflecting the structural remodeling function of MMPs in the progression of many epithelial cancers, such as lung cancer, breast cancer, and pancreatic cancer [11, 12].

Meanwhile, cell-to-cell activity residing in the TME is constantly reshaping ECM, and these cells are affected by the signals provide by ECM itself [13]. Cytotoxic immune cells, which mainly conduct immune elimination, are incapable of stifling tumor cells in the remodeled ECM because of the formation of an immunosuppressive environment. Thus, ECM acts as a rock-solid shield protecting tumor survival and progression. However, there is no shield that cannot be destroyed. ECM can be a double-edged sword; while it stimulates tumor progression, it can also be a sally port to antitumor therapy. In this paper, we discuss the key role of ECM remodeling in the TME and its interaction with tumor cells, including the molecular composition of ECM and the impact of ECM remodeling on the occurrence and development of malignant tumors. We also highlight the impact of ECM remodeling on therapeutic resistance and potential therapeutic targets.

## Molecular composition of ECM

The ECM is described as a collection of exocrine molecules that provide structural and biochemical support for the surrounding cells [14]. Similar to the significance of soil composition to plants, the ECM is the basic condition to provide adherable environment for cell proliferation and survival. From the perspective of molecular composition, the ECM mainly includes structural proteins (such as collagen and elastin), glycosaminoglycan, proteoglycan, and adhesion proteins (such as fibronectin and laminin). At the structural level, the ECM contains interstitial connective tissue matrix and basement membrane [15, 16]. The basic functions of the ECM include sustaining cell proliferation, differentiation and maintenance of tissue homeostasis [17]. Whereas, different kinds of organs or tissues have their own specific composition of ECMs. For example, loose connective tissue ECM is made up of reticular fibers and ground substances, and bone ECM contains collagen fibers and bone minerals, and the ECM for circulating blood cells is plasma. ECMs with different characteristics and compositions can play a role in many mechanisms. Various carbohydrate-rich polysaccharides and protein-rich fibers play an important role in maintaining tissue homeostasis and hydration [18]. The physical pressure on the ECM is controlled by the interspace composed of fibrils and the compression and buffering activities of polysaccharide gel [19, 20]. In normal tissues, the sequentially regulated ECM acts as a signal library and provides anchorage points and architectural definitions for mechanical sensing [21]. Whereas in the TME, ECM structures are reconstituted and intercellular signals are disrupted [22]. Therefore, better understanding the composition and structural characteristics of ECM remodeling in cancer is critical for discovering therapeutic targets and diagnostic markers [23].

## Multiple factors mediate ECM remodeling

### Hypoxia and CAFs

Hypoxia is a common feature of TME. Indeed, hypoxia is the result of unlimited expanding of tumor tissue and increasing requirement of nutrients including oxygen. It can weaken the function of cytotoxic T lymphocytes (CTLs) and attract regulatory T cells (Tregs), thereby reducing the immunogenicity of tumors and enhancing the invasive clonal expansion of heterogeneous tumor cells [24]. With the advancing of tumor progression, the establishment of hypoxic microenvironment can promote hypoxia-inducible factor 1 (HIF-1) activation-dependent signal transduction and help tumor cells and stromal cells adapting to surrounding hypoxia conditions, thus supporting tumor progression [25]. Hypoxia-induced increased expression level of several ECM remodeling enzymes such as LOX and collagen prolyl 4-hydroxylase (C-P4H) has been reported to mediate modification on collagens and promote tumor progression [26–28].

Endogenous fibroblasts overexpressing HIF-1α can promote tumor growth both in vivo and in vitro [29]. Nevertheless, another research suggested that chronic hypoxia plays dual role on cancer-associated fibroblasts (CAFs) function in a HIF-1α dependent manner [30]. On the one hand, HIF-1α can promote the carcinogenic effects of CAFs, such as promoting tumor growth, while block other carcinogenic functions, such as tumor invasion and metastasis [30]. Significantly, CAFs are main “architects” to mediate ECM remodeling and cause ECM stiffness and degradation [31]. Rising evidence emphasize different role of specific subtypes of CAFs in pancreatic cancer [32, 33]. Even a proinflammation role of hypoxia-induced CAFs has been reported recently [34]. Therefore, the role of Hypoxia-mediated CAFs in the whole process of tumor development needs to be further revealed.

### ECM remodeling enzymes

In the development of various organs, the LOX family of enzymes trigger extracellular collagen crosslinking, which also contributes to the formation of ECM stiffness in malignancy, in a hypoxic environment [35, 36]. During hypoxia, key target genes regulated by HIF transcription factors include LOX, LOXL2 and LOXL4. These transcription factors are intricate in collagen crosslinking and are one of the key factors leading to tumor fibrosis [37]. In general, hypoxic signal transduction may be involved throughout tumor progression and contribute to ECM remodeling in the tumor microenvironment. Besides of LOX and LOXLs, WISPs can also mediate collagen I linearization to control cancer metastasis. WISP1 fuels the linearization and metastasis and is overexpressed in cancer cells, while WISP2 against the process but its expression level is suppressed [38]. However, a recent report highlights that CAFs-derived WISP1 can hampers lung metastasis [39]. It is considered that the derivation of WISP1 may play the critical role.

MMPs are a group of zinc-dependent endopeptidases that bind to various ECM proteins and are one of the key enzymes for connective tissue remodeling [40, 41]. A big family contains more than 30 kinds of MMP have been identified since the MMPs’ role as collagen hydrolase was unveiled [42, 43]. MMP-2, -3, -9 and -14 are overexpressed and associated with ECM remodeling in a variety of malignant tumors [44, 45]. In the process of tumor progression, MMP-2 and MMP-9 can mediate the invasion of tumor cells into the basement membrane through the degradation of collagen IV, thus resulting in tumor metastasis and diffusion [46]. Besides, collagen degradation is an important mechanism for remodeling the biomechanical properties of ECM. Immunocytes recruited by LOX at the pre-transfer site degraded collagen IV through high expression of MMP-2, then promote the formation of pre-transfer niche by MMPs [47]. Conversely, reduced activity of MMPs inhibits pulmonary vascular permeability and limits the infiltration of immunocytes in the lung prior to metastasis [48]. Similarly, MMP-14-induced collagen dissolution around tumor cells is also one of the critical factors for cell invasion and migration [49]. MMP-14 produced by the tip cells of polarized multicellular masses degrades interstitial collagen, resulting in a locus that is a key pathway for cell invasion [50, 51]. Besides of ECM degradation, the binding of MMP-9 to α4β1 integrin induces several intracellular signaling to promote anti-apoptotic pathway and metastatic pathway in cancer cells, suggesting another pivotal mechanism of MMPs-induced tumor progression [52, 53].

Heparan sulfate proteoglycans (HSPGs) are one of major component of ECM and can regulate cell behavior and maintain stromal structure stability by binding and releasing many signaling molecules, such as interleukin-8 (IL-8), fibroblast growth factors (FGFs) and vascular endothelial growth factor (VEGF) [54, 55]. Heparinase is an essentially endo-β-D-glucuronidase, which can degrade HSPGs to produce low molecular weight fragments [56]. High expression of heparinase has been detected in a variety of tumor patients and is significantly associated with poor prognosis in patients with head and neck squamous cell carcinoma [57], breast cancer [58], and gastric cancer [59]. Physiologically, heparinase is produced by keratinocytes, platelets, placental trophoblast cells, and white blood cells (including mast cells) [60]. In the tumor microenvironment, heparinase drives the cleavage of HSPGs to enhance the availability of various secretory factors, leading to tumor angiogenesis, and promoting cell invasion and migration [61–63]. Heparinase can also degrade perlecan and syndecan-1 (Sdc-1/CD138) to mediate tumor cell growth and invasion [64]. Interestingly, heparinase plays a crucial role in the invasion of natural killer (NK) cells into dense tumor ECM, thereby resisting tumor progression and metastasis, emphasizing that the ECM is a barrier for both tumor cells and immunocytes [65].

Besides of enzymes discussed above, more tumor-associated ECM remodeling enzymes have been unveiled. These enzymes show significant role in boosting tumor progression and can be considered as potential candidates for anticancer therapy.

### Myeloid cells

Myeloid cells are the main hematopoietic cells in the human body, which are differentiated from hematopoietic stem cells [66]. Myeloid cells are involved in ECM remodeling in varying degrees, and produce both ECM remodeling enzymes/mediators and ECM molecules directly. In the tumor microenvironment, MMPs are primarily derived from myeloid cells and are involved in ECM collagen remodeling [67]. Stromal cell protein SPARC is a matrix regulator and collagen chaperone [68]. SPARC-deficient microenvironment in breast cancer reduced primary tumor growth and lung metastasis, possibly due to the macrophages with SPARC-deficiency and unable to support stroma formation and collagen deposition [69, 70]. These studies suggest that macrophages may be the source of ECM-related proteins. Transcriptomic and proteomic analyses of these cells have demonstrated that tumor-associated macrophages (TAMs) are one of the sources of ECM molecules and process collagen synthesis, stability, assembly and cross-linking [71, 72]. Abnormal collagen fiber deposition was found in colorectal cancer grown in macrophage-deficient mice [73]. Precisely, TAMs can regulate collagen production by stimulating CAFs [73, 74]. In pancreatic cancer, TAM-derived C-X-C motif chemokine ligand 3 (CXCL3) targets CAFs’ C-X-C motif chemokine receptor 2 (CCR2) to mediate CAF-myofibroblasts (myCAF) transition, subsequent type III collagen generation and tumor metastasis [75]. Intriguingly, single cell analysis uncovered a specific TAM-CAFs transition in lung cancer, highlights the consistent interaction between TAMs and CAFs [76]. The precise introduction of TAMs in ECM remodeling will be discussed later.

### Microbiome

With the in-depth study of tumor microenvironment, the role of microbiome in tumor ECM remodeling cannot be ignored [77, 78]. There are several sets of evidence that the microbiome is actively involved in creating the tumor microenvironment and interacting with multiple elements [79, 80]. Representative research found out that subcutaneous injection of *M. hyorhinis* in mice can develop resistance of pancreatic ductal adenocarcinoma (PDAC) to gemcitabine, and further investigation unveiled microbiome prevalence (especially γ-proteobacteri*a*) in approximately 75% of human PDAC clinical samples [81]. Similarly, other researchers have reported the presence of different types of microbiotas in samples from multiple malignancies [82, 83]. Clinical trials about chemotherapeutic agents, cyclophosphamide and oxaliplatin, have shown that reactive oxygen species (ROS) produced by the microbiome in the tumor microenvironment contributes to better chemotherapeutic efficacy [84]. The efficacy of these drugs was significantly reduced in immunocompetent mice compared to germ-free mice, along with significantly reduced tumor clearance and immune activation, highlighting the critical role of symbiotic bacteria in the regulation of the host immune system [85].

Positive interactions between tumor cells and the microbiome increase the likelihood of ECM remodeling leading to cancer niche formation, tumor progression, and drug resistance. A variety of bacterial enzymes (such as collagenase, elastase and hyaluronidase) are known to degrade host ECM [86, 87]. Studies have shown that in bladder cancer, the interaction between the host bacterial population and ECM components regulates the main composition of the microenvironment, thus determining tumor growth and metastasis [88]. In addition, the microbiome can induce intestinal fibrosis by triggering host immune cells [89]. Taken together, all these results suggest that the microbiome can influence host ECM and its homeostasis, but the details of these interactions have not been elucidated and extensive further studies are needed to accurately elucidate the molecular and signal cascades involved.

## ECM remodeling and cancer proliferation

Unlimited expanding of tumor tissues results in increasing risk of heterogeneity and severe local vicinity which abrogates the infiltration of anti-tumor agent and cells in TME. It’s worth noting that the sum of bulk acellular and cellular components in TME, specifically cells with accelerated cell cycle and/or suppressed cell death regulated by ECM, leads to the uncontrolled expanding of cancer. However, different composition of ECM determines various fates of cell proliferation. Phenomena from the radiation-induced ECM regulation on cell cycle arrest/progression of both malignancy [90, 91] and fibroblasts [92] underpins the opinion. Nevertheless, most studies indicated that acellular components secreted by depositional cells show the potential to participate in oncogenic progression, or suppress cancer growth to transform tumor cells into a quiescent state, which endowing cells with stem-like characteristics and proliferative potential, for against stresses during growth and metastasis. For instance, the role of type III collagen in maintaining tumor dormancy to form a metastatic niche has been unveiled [93]. Therefore, ECM seems like a specific reaction pool filled with signals of mechano-transduction and bioactive molecules transduced from cancer and stromal cells to regulate tumor tissue proliferation, and it is emergency to unveil the program of ECM remodeling and identify specific therapeutic target for cancer elimination [94].

### Receptors and signaling pathways: eyes blinded by mirage

**Fig. 1:**
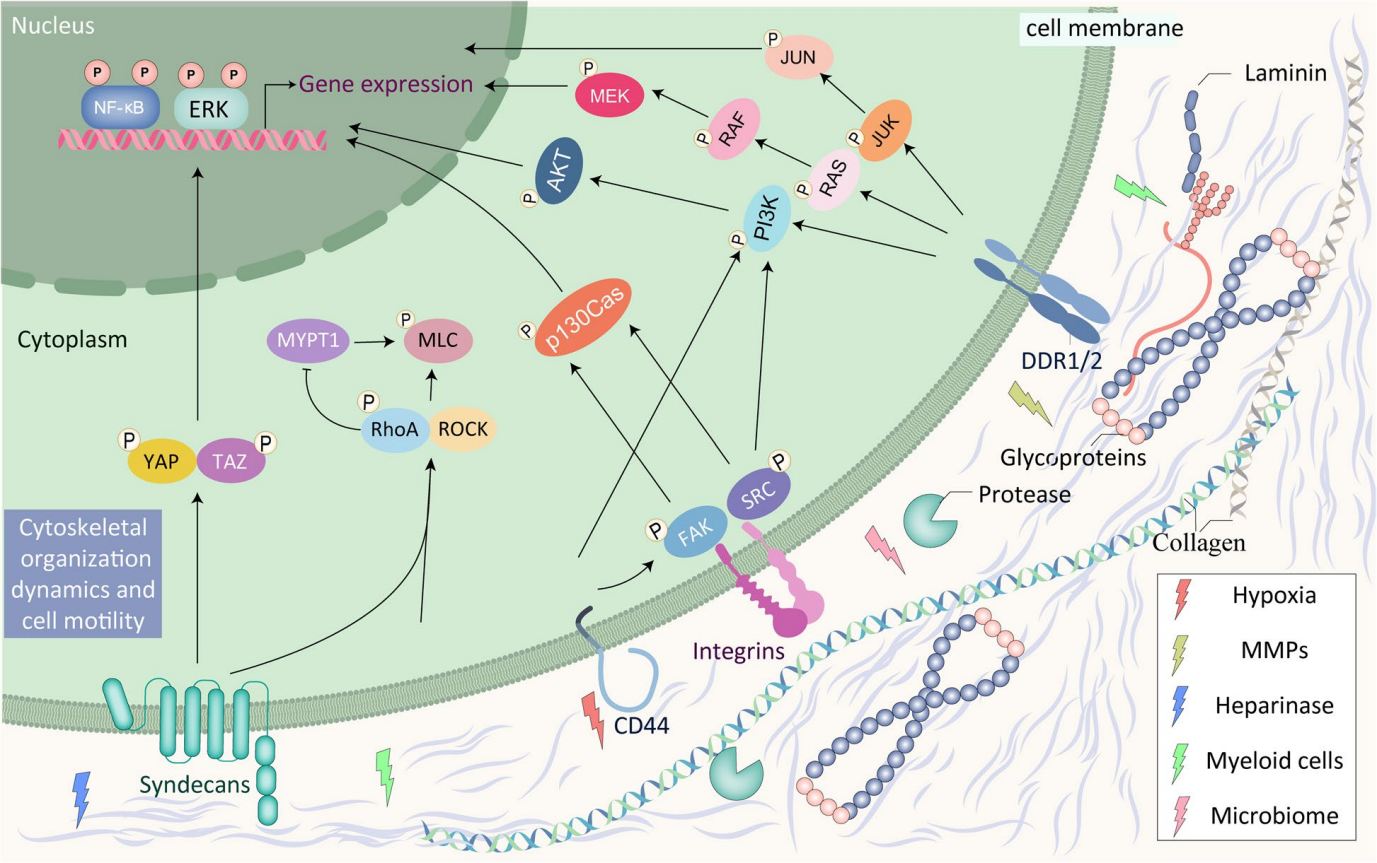
Receptors for cell-ECM interaction. Matrix changes modulate intracellular signaling in cancer, changes in the extracellular matrix regulate many intracellular signaling pathways. However, the illustration only summarizes familiar receptors in cell-ECM interaction, such as integrins, DDRs, CD44 and syndecans. Other receptors and regulatory networks are precisely introduced in the context

The activation of cell cycle signals is critical for tumor proliferation. ECM can activate or inhibit intracellular signal transduction process, thus regulating cell biological behavior. The most prominent transduction pathway downstream of ECM signals is direct transduction, which occurs via classical transmembrane gated proteins such as integrins [95]. Dimerization of integrin subunits (including α chain and β chain) triggers phosphorylation of the focal adhesion kinase (FAK)/Src pathway, leading to increased cell adhesion and migration [96]. Substantial transformation of cell behavior occurs due to activation of intermediary pathways downstream of FAK/Src activation, such as the extracellular signal-regulated kinase 2 (ERK2)/mitogen-activated protein kinase (MAPK) cascade, and small guanosine triphosphatase (GTPase) (such as β-catenin pathway, Rac and Rho) (Fig. 1) [97–99]. Attentionally, specific integrin β subunits are critical for cancer cell proliferation in different aspects. For instance, β1 integrin alteration physiologically maintains mammary gland proliferation and is associated with accelerated cell cycle and temsirolimus resistance in bladder cancer [100, 101]. By contrast, β3 integrin is necessary for stem-like tumor-repopulating cell (TRC) dormancy [102], and CD90-inhibited anchorage-independent growth in cancer stem cells (CSCs) [103]. Thus, β1 integrin inclines to accelerate cell cycle, whereas β3 integrin prefer to stemness.

Hyaluronan/hyaluronic acid (HA) is a member of glycosaminoglycans and prevalently overproduced by cancer and stromal cells. The interaction of HA and membrane receptor CD44 or Toll-like receptor 4 (TLR-4) sustains proliferation of cancer and the formation of fibrosis [104–106]. HA/CD44 stimulates epidermal growth factor receptor (EGFR) singling pathway [107], whereas HA/TLR4 is associated with the activation of nuclear factor-kappaB (NF-κB) pathway [106, 108]. Receptor for hyaluronan-mediated motility (RHAMM, or CD168) is another HA-specific receptor that mediates inflammation and tumor progression [109]. Physiologically, hyaluronan synthase 2 (HAS2)-dependent HA/CD44/RHAMM pathway is critical for mammary gland morphogenesis [110]. In glioblastoma, the negative association between the concentration of extracellular HA and the efficacy of EGFR inhibitor indicates that HA is a main signal factor to stimulate cell proliferation [111]. Rho-GTPase is the primary downstream pathway to response HA/CD44 activation, and inhibition of Rho-associated coiled-coil containing kinase (ROCK) results in elevated CD44 expression and maintaining of CSCs [112, 113]. Both Rho-GTPase and EGFR could stimulate phosphoinositide 3-kinase (PI3K)/Akt pathway to sustain proliferation in cancer cells [114]. Attentionally, the function of HA in pro- or anti-oncogenesis is determined by its’ molecular mass [115–117]. Thus, the identification of HA molecular mass in cancer is critical.

Syndecans are cell-surface heparan sulfate proteoglycans known to play a role in cell adhesion, migration, and binding of growth factors. A canonical signaling about syndecans in cancer is heparinase/syndecan-1 pathway [118]. Heparinase stimulates syndecan-1 expression, cleavage and shedding to promote fibrillar collagen deposition and tumor growth [119–121]. Shedding syndecan-1 could combine with VEGF and adhere to ECM, then induce invasion and angiogenesis in melanoma [121]. While depletion of syndecan-1 in colon cancer cell line in turn stimulates heparinase expression and retain cells into a stem-like state [122]. Furthermore, previous explorations have identified that tenascin-C (TNC) interacts with syndecan-4 and blocks integrin/syndecan complex to mediates cell-fibronectin adhesion, which sustains proliferation and induces angiogenesis in cancer [123–125]. After that, more subtypes of syndecans have been identified to be associated with progression of cancer [126, 127].

Stromal cells in ECM such as CAFs play a significant role in supporting cancer cell proliferation. A colorectal patient-derived organoid (PDO) model shows that solely CAFs are sufficient to support cell proliferation with the absence of conventional PDO-associated growth factor, highlight the CAFs’ pro-oncogenic role in cancer culture maintenance [128]. In a 3D model of PDAC, pancreatic stellate cells (PSCs) could play the same role [129]. A 3D-bioprinting culture of glioblastoma which contains cancer cells, endothelial cells, and hyaluronic acid derivatives has proved the significance of ECM stiffness on the variation of gene transcription program and interaction between cancer and endothelial cells [130]. Moreover, high expression of syndecan-1 in CAFs correlated with tumor progression in specific cancer types from breast, colon, prostate, ovary and lung [131–134]. Matrix enzymes and ECM components secreted from stromal cell are critical for cancer cell proliferation, while cancer secrets soluble growth factors sustaining stromal cells survival and ECM generation in turn [135, 136]. Therefore, the interaction between cancer cells and stromal cells controls ECM remodeling leading to the proliferation of cancer, whereas the expanding tumor tissue will face some troubles, especially under the overseeing of pathways controlling organ size.

### Escape from tissue size surveillance

Hippo signaling pathway is a key regulator of tissue growth and organ size control when facing mechanosensory pressure and increasing cell adhesion. Though crowded space caused by tumor proliferation and desmoplasia may result in enhanced mechano-transduction and intercellular contact, cancer cells present the tenacity to adapt the pressure from ECM and maintain proliferation. Abnormal activation of Hippo core factors Yes1 associated transcriptional regulator (YAP) and WW domain containing transcription regulator (TAZ, or WWTR1) sustain cell proliferation and is prevalently detected in various cancers, implying the success adaption of cancer to crowed environment [137, 138]. Integrins and downstream pathways are main signal traducers for YAP/TAZ activation [139]. Among them, canonical Rho GTPases senses signals from ECM and regulates YAP/TAZ activation, while dual role of Rho GTPases in tumor progression is dependent on their expression and mutation states [140].

**Fig. 2:**
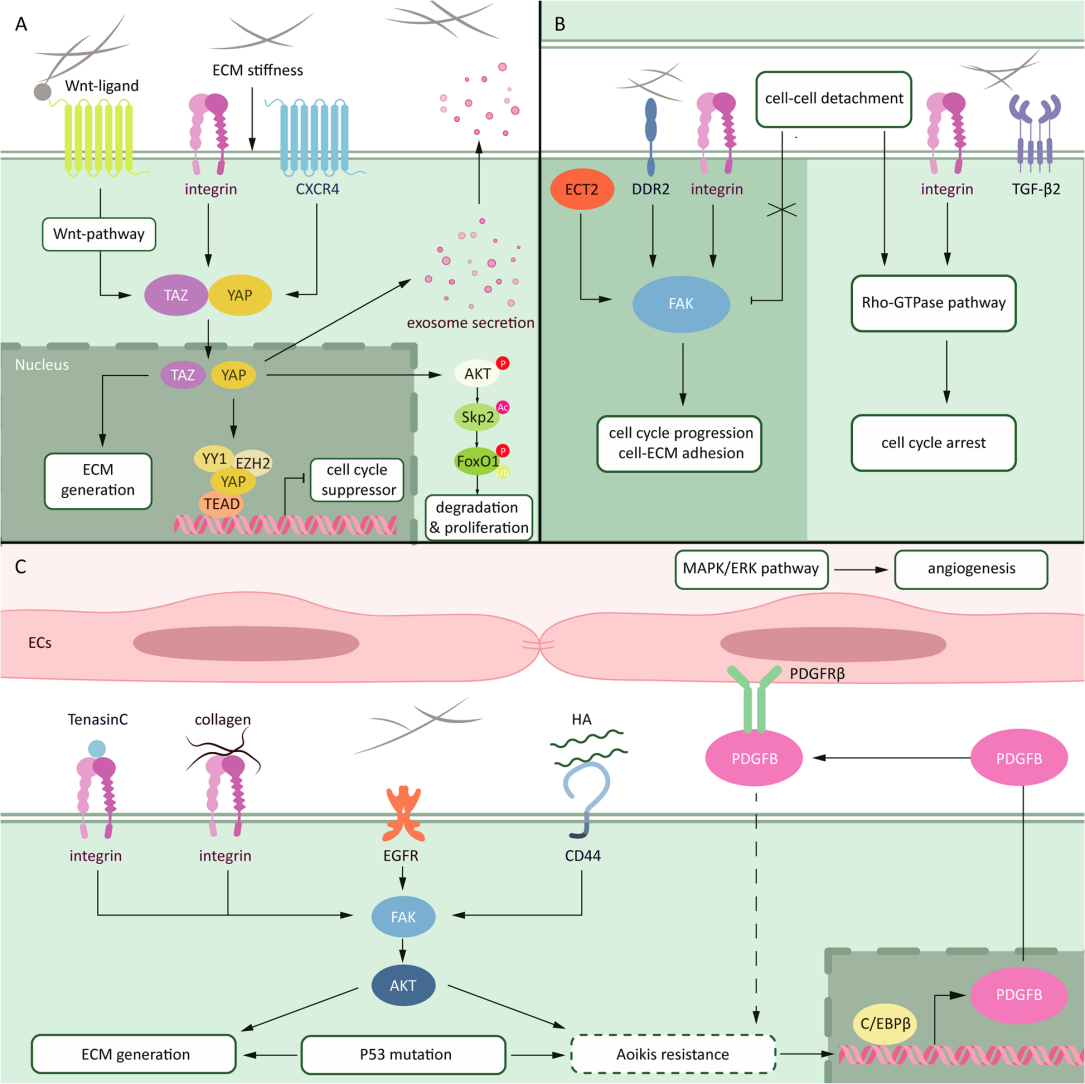
ECM role in proliferation, cell cycle arrest and anoikis resistance. The changes in ECM proteins, especially the crosslink of collagen, enhance the stiffness of ECM. Receptors sense the mechanic signal form ECM and activates downstream pathways including FAK and YAP/TAZ to induce cell cycle progression and cell-ECM adhesion (**A**). Moreover, ECM support detachment cell survival, even induce cell cycle arrest in a quiescent state to decrease energy consumption and resist to anoikis (**B**). Several components support the acquirement of anoikis resistance such as tenascin-C, collagen, HA and PDGFB (C)

Other receptors identified recently such as CXCR4 in hepatoma also senses stiffness of ECM and activates YAP/TAZ activation [141]. Moreover, dysregulation of YAP/TAZ co-transcriptional function triggers and impairs autophagy sensitivity to contact inhibition via absent F-actin expression [142], while ECM stiffness stimulates YAP/TAZ activation and exosome secretion to enhance cell mobility [143]. Besides manchanotransduction, Wnt ligand-linked collagen culture is sufficient to induce YAP/TAZ activation and support cellular reprogramming [144], in accord with previous discovery of Wnt-YAP/TAZ pathway [145, 146]. It’s worth noting that roles of YAP and TAZ in cell cycle regulation are considered different. In non-small cell lung cancer (NSCLC), YAP favors cell cycle progression while TAZ is preferentially associated with ECM organization [147]. Role of TAZ in ECM remodeling has been described in fibroblasts and adipocytes [148, 149]. Moreover, YAP increases nucleic accumulation of P27, a cell cycle suppressor by acetylating and nucleic-exporting S-phase kinase associated protein 2 (SKP2) via Akt activation to sustain cytoplasm retention of SKP2 and to inhibit mitosis of cancer cells, which causes polyploidy formation [150]. While accumulation of cytoplasmic SKP2 then triggers ubiquitination of forkhead box protein O1 (FOXO1) and then stimulates cell proliferation [150]. Nevertheless, another research found out that nuclear YAP inhibits P27 expression in a transcriptional pattern [151]. These results indicate that YAP activation is a primary factor to induce cell proliferation under the surveillance of Hippo pathway (Fig. 2A). Compared with malignant cells, YAP activation in PSCs triggers the secretion of SPARC, an ECM protein, to suppress tumor growth [152]. Importantly, ECM is essential for reprogramming of normal cells into premalignancy, and YAP/TAZ is responsible for the transformation induced by ECM [153]. The crosstalk of YAP/TAZ pathway between cancer cells and stromal cells also provides novel view in ECM-induced tumor progression [154]. Thus, it’s critical to unveil the precise function of YAP and TAZ in ECM-induced tumor progression.

### Cell cycle progression and quiescent: the golden mean

With tumor progression, epithelial-mesenchymal transition (EMT) transforms cells into heterogeneity which favors invasion and metastasis. Some cells lose their intercellular adhesion and detached from tumor tissue in a timepoint. However, cell adhesion is still necessary for survival, especially for detached cancer cells with insufficient preparation. Intracellular contact provides basal signal for cell survival and loss of cell adhesion triggers cells death. Physiological tissue stiffness, which causes decreased cell adhesion, suppresses cell cycle via inhibiting FAK signal responding and downstream Rac-dependent Cyclin D1 expression [155, 156]. However, high matrix density caused by cancer provides components for cell adhesion and stimulates vital survival signaling against detachment. Pathologic ECM induces FAK and ERK signaling pathway activation to promote cell proliferation in breast cancer [157] and gastric cancer [158]. Within ECM, collagen I sustains CSCs renewal and tumor initiation via the activation of FAK in pancreatic cancer [159]. Moreover, epithelial cell transforming sequence 2 (ECT2)/FAK and collagen type X alpha 1(COL10A1)/discoid protein domain receptor 2 (DDR2)/FAK pathways contribute to the proliferation and adhesion on ECM of lung adenocarcinoma cells [160, 161]. Thus, ECM components provide signals to activate FAK pathway and sustain cell proliferation under the situation of detachment, which is critical for pre-metastasis state formation of cancer (Fig. 2B).

Some therapies are introduced to target ECM-induced proliferation which may hamper cancer cell transformation into pre-metastasis state. Homologous esophageal ECM treatment hinders malignant proliferation and suggests a suppression of cell cycle in neoplastic esophageal cells [162]. Moreover, synthetic materials are considered to mimic the variation of ECM and observe changes in cell cycle, such as C_60_ nanofilm [163]. Identification of critical cell cycle factors may also contribute to the discovery of ECM-based anti-tumor treatment. Cyclin-dependent kinases 4 and 6 (CDK4/6) inhibitor, which targets the combination of cyclin D1 and CDK4/6 to suppress cell cycle, shows extensive influence in inhibiting ECM deposition and oncogenic properties [164]. Besides Cyclin D1, CDK1 recently has been recognized as a pivotal cell cycle regulator which response to cell adhesion and has a profound association with cancer progression [165–167]. However, the precise mechanism of CDK1 in responding to pro-oncogenic ECM is still unclear.

By the way, cell cycle arrest doesn’t present a low risk of tumor progression. The state of quiescence featured by reversible proliferative arrest reserves the capacity to reenter the cell cycle upon receiving an appropriate stimulus [168]. When facing therapeutic treatment or disseminated from the primary tumor tissue during metastasis, cancer cells with quiescent state are able to survival under these pressures. Before obvious formation of distant metastasis, systemic dissemination of quiescent tumor cells has been existed depending on normal ECM-cell adhesion-induced syndecan-1 activation [169]. Quiescent cancer cells with the potential of recurrence form a comfortable environment of fibrillar fibronectin matrix via integrin, ROCK and transforming growth factor β2 (TGF-β2) pathways, whereas an outgrowth would be started by MMP-2-mediated ECM degradation in breast cancer [170]. In melanoma, ECM stiffness triggers translocation of Cdc42, a transcription factor belongs to Rho-GTPase, then upregulating ten-eleven translocation 2 (Tet2) expression which resulting in induction of quiescent via decreasing expression of p21 and p27 [102]. A gene signature analysis based on glioblastoma PDO model supports the correlation between quiescent state and hypoxia/TGF-β-dependent ECM [171]. Hydrogel models cultured by cancer cell lines from different organ demonstrate various quiescent dynamic, which implying a significant of organ specific ECM in the maintenance of quiescent state [172]. Changes in chemical and mechanical properties in hydrogel culture determines balance between quiescence and reactivation [173]. These phenomena highlight that stiffness and biomolecules in ECM are both critical for quiescent state sustaining. Herein, tumor with enhanced proliferation and necessary quiescence triggered by ECM becomes invasive and dangerous.

### Anoikis resistance: survival from detachment

Anoikis is a property of programmed cell death responding to the detachment between cell and ECM. Anoikis is pivotal in normal development and tissue homeostasis, whereas anoikis resistance is essential in cancer invasiveness and metastasis [174]. As described previously, the activation of FAK signaling pathway plays the pivotal role in sustaining cancer cell proliferation from detachment. Signal receptors such as integrins are critical for FAK activation. While inhibition of integrin can mimic the situation of cell-ECM detachment and blocking integrin suggests a reasonable treatment to trigger anoikis and tumor suppression. Arg-Gly-Asp (RGD) peptide is designed to specific target and block integrins, and shows the stimulation of anoikis in glioma cancer stem cells [175]. Expressing β3 integrin in cancer cells is essential for distant metastasis in breast cancer [176]. Specific RGD targeting β3 integrin could inhibit adhesion and tumor-derived small extracellular vesicles absorption in normal breast cells [177], it also favors anti-apoptosis and autophagy in breast cancer cells [178]. Whereas in esophageal squamous cell carcinoma, indomethacin driving β3 integrin blocking results in reduced tumor growth and the potential of metastasis [179]. The paradoxical phenomenon may due to the anti-tumor immune response triggered by indomethacin.

EGFR pathway is strong associated with detachment induced anoikis. Targeting EGFR is effective for anoikis suppression [180]. For instance, α2β1/α5β1 integrin/EGFR pathway supports cell survival and stimulates anoikis resistance in colon carcinoma [181]. Pre-mRNA splicing factor 4 kinase (PRP4K), a pre-mRNA splicing enzyme, decreases degradation of EGFR during detachment and induces anoikis resistance [182]. Moreover, when PDAC treated with ECM depletion agent FAK inhibitor, the abnormal expression of signal transducer and activator of transcription 3 (STAT3), downstream of EGFR, hampers the function of FAK inhibitor and leads to drug resistance [183]. Furthermore, syndecan binding protein melanoma differentiation associated gene-9 (MDA-9, or syntenin), sustains EGFR signal and protective autophagy against anoikis in glioma CSCs [184]. Lung fibroblast expressed syndecan-1 promotes breast cancer lung metastasis, implying the pro-oncogenic role of this glycoprotein in ECM [185]. Syndecan-4 also participates in the progression of anoikis resistance [186, 187]. Intriguingly. inhibition of EGFR triggers suppression of EMT, Notch pathway and syndecan-1 expression via ERβ pathway, suggests the pivotal role of EGFR in anoikis resistance [188].

Components within ECM sustain cell survival against detachment induced anoikis. Collagens such as collagen I/β1 integrin in gastric cancer [189], collagen XIII/β1 integrin in breast cancer [190], COL11A1/Akt/Cyclic AMP response-element binding protein (CREB) in PDAC [191], collagen IV/integrin in hepatoma [192], support anoikis resistance via B-cell lymphoma (BCL) family proteins and downstream pathways. Attentionally, acid stress in local environment is associated with the function of collagens on tumor cells [193]. Suppression of phosphatases also contributes to the inhibition of collagen generation, highlights the critical role of posttranslational modification of collagen [194]. HA/CD44 also contributes to the activation of Akt pathway, which is responsible to collagen-induced anoikis resistance [195, 196]. Compared to the activation of AMP-activated protein kinase (AMPK) pathway in circulating cancer cells, Akt activation is prevalently detected in primary and distant metastatic cancer cells, and the negative feedback loop between AMPK and Akt implying the ability of adaptation of cancer cells to ECM [197]. Besides, platelet-derived growth factor-BB (PDGFB) secretion from anoikis resistant gastric cancer cells constructs a C/EBPβ-PDGFB-PDGFRβ-MAPK feedback loop with vascular ECs, which supports angiogenesis and metastasis in gastric cancer [198]. Tumor-secreted PDGF also stimulates CAFs to facilitate ECM stiffness [199]. Additionally, α11 integrin/PDGFRβ^+^ CAFs respond to the stimulation and promotes invasion and metastasis via c-Jun N-terminal kinase (JNK)/TNC in breast cancer [200]. Under the chemotherapy, JNK pathway activation triggers osteopotin (or SPP-1) and TNC secretion, then induces chemoresistance and metastasis in breast cancer [201]. Intriguingly, TNC-derived peptide TNIIIA2 confers anoikis resistance and PDGFRβ activation in an α5β1 integrin-dependent manner, suggests a possible positive feedback loop between cancer and CAFs [202, 203]. With the stimulating of ECM components, cancer cells are advantaged to survival against anoikis (Fig. 2C).

Indeed, intercellular and intracellular elements take together trigger anoikis resistance. P53 is a pivotal factor to mediate cell apoptosis, and the existence of gene mutation of P53 is prevalent in most cancer cells [204]. Physiologically, losing of cell adhesion would trigger P53-mediated apoptosis to sustain tissue homeostasis. ECM components provide the loci for cell adhesion and overcome the P53 mediated apoptosis in glioblastoma [205]. While the treatment of FAK inhibitor, which results in ECM depletion, in overcoming chemoresistance depends on the activation of P53 signaling pathway [206]. Nevertheless, P53 mutation plays a pivotal role in anoikis resistance against losing adhesion signals [207]. When β1 integrin is blocked, mutation of P53 in cancer fails to response to the absence of β1 integrin signal therefore bypasses apoptosis [208]. Moreover, P53 mutation itself links to ECM remodeling and mediates cancer progression. For example, P53 mutation is necessary for HIF-1 constructed ECM [209]. It also increases podocalyxin sorting into exosomes and induce ECM deposition [210]. Mechanically, mut-p53/HIF1α/miR-30d axis triggers tubulo-vesiculation of the Golgi apparatus and promotes ECM deposition and remodeling [211]. Thus, adequate preparation for the distant metastasis of malignancy has been prepared under the circumstances of ECM remodeling (Fig. 2). These variations link anoikis with metastasis of cancer cells, suggest that anoikis is prerequisite to cancer invasion.

## ECM remodeling and metastasis in cancer

ECM remodeling constructs the architectural and bioactive environment to support cancer invasion and metastasis. Tumor surrounded by collagen-rich circumstances not only faces the pressure from ECM stiffness, but also receives stimulators from other components within ECM. When tumor cells overcome the detachment-induced anoikis, further variations on the phenotypes of cancer are needed for distant metastasis. These characterizations including EMT for basic ability to induce invasion, collagen orientation for metastatic architectural construction, matrix-related enzymes to facilitates ECM remodeling, metabolism preparation to support cell metastasis, secretion of ECM components to provide cell-ECM adhesion and drivers mediates metastasis from primary loci to intravasation. All features are essential for ECM-mediated tumor invasion and metastasis, and the understanding of this profound network may contribute to the anti-tumor therapy targeting ECM. Some ECM components promote cancer progression doesn’t mentioned below are listed in Table 1.

**Table 1:** Role of other ECM proteins in tumor progression

Cancer	Proteins	Results	References
Pancreatic cancer	Fibronectin	Fibronectin determines SPARC function to control proliferation and anti-apoptosis	[212]
Prostate cancer	Plectin	Plectin Knock-down impairs cell growth and metastasis	[213]
Breast cancer	FGF2	FGF2 promotes ER/Cyclin D1 signaling and endocrinotherapy resistance	[214]
	Fibulins	Estradiol indued fibulin-1 inhibits fibronectin induced motility of cancer	[215]
Glioblastoma	Collagen I and fibronectin	3D culture model stimulates tumor progression via PI3K and CDC42 pathway	[216]
	Fibulin-3	Inhibition of fibulin-3 suppresses activation of ADAM17/Notch/NK-κB pathway and tumor progression	[217]
Hepatoma	Periostin	Autocrine loop of periostin/integin/FAK/STAT3/perison in hepatic stellate cell promotes hepatoma cell proliferation via paracrine stimuli	[218]
	Epimorphin	Cancer secretes epimorphin promotes malignant invasion and metastasis	[219]
	Laminin-332	Laminin 332 antibody targeting matrix binding domain suppress tumor progression	[220]
		Laminin 332 sustains stem state and chemoresistance via phosphorylating mTOR	[221]
Colorectal cancer	MGP	MGP expression favors oxaliplatin resistance	[222]
	Periostin	Interaction loop between fibroblast secreted periostin and cancer derived IL-6 contributes to the progression of colitis-associated colorectal cancer	[223]
	Fibulins	Fibulin-5 inhibits TRPV1 to induce cell apoptosis via ROS/MAPK and Akt pathways	[224]
	Fibrinogen	Fibrinogen actives FAK to suppress P53 and sustain cell proliferation and anti-apoptosis	[225]
	HAPLN1	HAPLN1 against TGF-β-induced COL1A1 expression and ECM remodeling	[226]
Gastric cancer	ECM1	ECM1 stimulate ITGB4/FAK/SOX2/HIF-1α to promote metastasis and glucose metabolism	[227]
	Lumican	CAFs derived Lumican induces tumor progression via β1-FAK pathway	[228]
Non-small cell lung cancer	Fibulins	Methylated FBLN2 inhibits tumor proliferation and cell adhesion via MAPK/ERK and AKT/mTOR pathways	[229]
	Hyaluronan	Hyaluronan-CD44/RHAMM mediates cell proliferation and anti-apoptosis	[104]
	Laminin	Laminin 5/integrin/FAK stimulates EGFR activation to promote anoikis resistance and invasion	[230]
Myeloma	Reelin	Reelin promotes cell proliferation and glycolysis via FAK/Syk/Akt/mTOR and STAT3 pathways	[231]
	Fibronectin	In vitro Coculture of cancer cell and fibronectin enhances MMP9 expression and MMP2 activation	[232]
Ovarian cancer	COL11A1	COL11A1-DDR2 stimulates Src-PI3K/Akt/NF-κB signaling to induce cisplatin resistance	[233]
Chondrosarcoma	OPN	Osteopontin stimulates MMP-9 expression via β3 integrin/FAK pathway to induce migration	[234]
Cutaneous squamous carcinoma	Fibulins	Fibulin-3 inhibits Akt signaling pathway and suppresses tumor progression	[235]
Cervical cancer	MFAP5	MFAP5 inhibition triggers cell cycle arrest and apoptosis	[236]

### ECM-induced EMT: ready for invasion

For epithelial cells, EMT is a critical process for tumor progression and metastasis [237]. EMT is featured by decreased expression of epithelial markers such as E-cadherin, and the upregulation of mesenchymal factors including N-cadherin, snail family transcriptional repressor (SNAIL), twist family basic helix-loop-helix transcription factor (TWIST), and other factors. These variations trigger mobility and invasion of cancer cells. Increasing number of researches focus on the function of ECM components on EMT in cancer cells. For instance, co-culture of high-metastatic breast cancer-derived ECM stimulates EMT in breast cancer [238]. When cellular components are eliminated, acellular scaffolds are sufficient to promote EMT and invasion of breast cancer [239, 240]. Specific chondroitin sulfate scaffold also stimulates EMT in prostate cancer [241]. A logical analysis based on EMT cellular regulatory network unveils ECM stiffness is a prerequisite for FAK/Src activation to stimulate SNAIL expression and intracellular adhesion via stimulating receptor-type tyrosine-protein phosphatases kappa (PTPRK) expression [242]. YAP may also play a significant role in mechano-regulated EMT. Wilms Tumor-1/YAP pathway suppresses E-cadherin expression and mediates cell–cell detachment, while YAP/Trio Rho guanine nucleotide exchange factor (TRIO)/Merlin mediated regulation of Rho GTPases, which promotes cell migration in renal cancer [243]. Gene-deficiency also confers cancer cells with EMT and metastasis via ECM generation, such as SET domain containing 2 (Setd2) in pancreatic cancer [244].

**Fig. 3:**
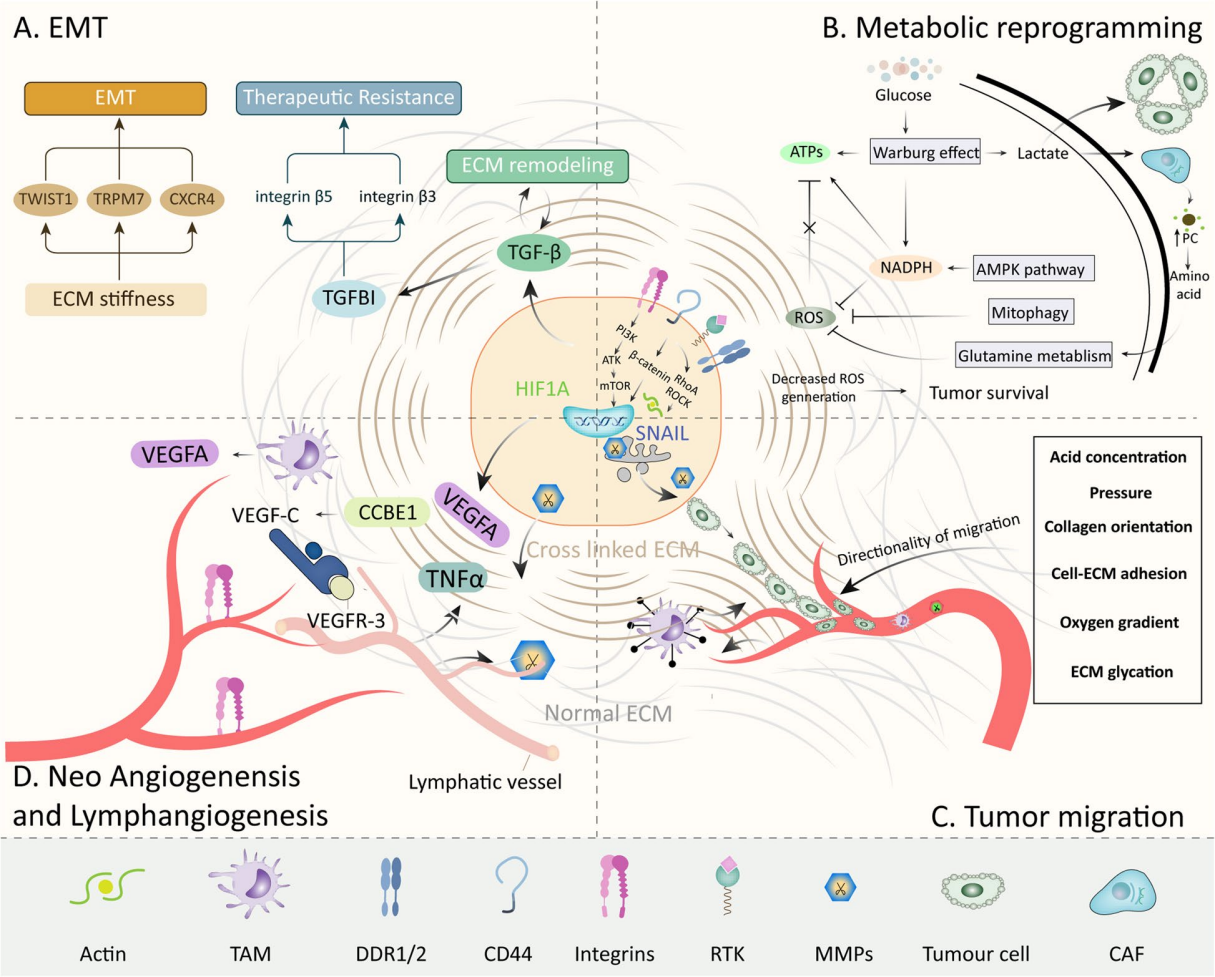
The process and regulatory network during tumor invasion and metastasis within ECM. The metastatic process of cancer cells is similar to a distant journey. EMT, which triggers decreased cell adhesion, confers the initiation of invasion (**A**). Then cancer cells with anoikis resistance show multiple changes in metabolic process to support energy requirement for cell survival and invasion (**B**). Thus, a gradient directionality constructed by several ECM components guides them to penetrate through the tissue for hematogenous and/or lymphatic metastasis (**C**), the activation of which has been stimulated by ECM components (**D**)

TGF-β is a primary inducer of EMT in cells whereas TGF-β signaling pathway plays dual function to promote tumor progression, or inhibits oncogenesis [245]. TGF-β promotes SRY-Box transcription factor 4 (SOX4) expression to mediate the cooperation between SOX4 and Kruppel-like factor 5 (KLF5) and induce subsequent pro-oncogenic EMT [246]. By contrast, small mothers against decapentaplegic protein 4 (SMAD4)-dependent EMT hinders the function and causes death in PDAC [246]. However, ECM-associated aberrant TGF-β-induced-EMT is prevalent observed in various cancers. RAS-responsive element binding protein 1 (RREB1) is a critical partner for TGF-β/SMAD-induced EMT, and fibrosis via downstream activating of SNAIL and other mesenchymal genes [247]. When treated with additional TGF-β, ovarian cancer cells demonstrate stimulated EMT and ECM remodeling [248]. In PDAC, TGF-β treatment triggers fibronectin and collagen I deposition, and metabolic variation which is responsible to metastasis [249]. ECM components such as the binding between tenascins and TGF-β isoforms [250] and EGF-domain of fibronectin fibrils [251] stimulate TGF-β signaling pathway and EMT process in normal cells, may potentially influence EMT in cancer cells **(**Fig. 3A).

In normal cells, TGF-β activation physiologically transforms myofibroblasts into fibroblast for pro-healing homeostasis sustaining [252]. However, the balance of this homeostasis will be destroyed under the regulation of cancer [32]. For instance, COL11A1 stimulates NK-κB/insulin-like growth factor binding protein 2 (IGFBP2) to induce TGF-β3 activation in ovarian CAFs to promote tumor growth [253]. PDAC cells induce TGF-β/SMAD5 activation in CAFs for branched-chain amino acids supplementation, which supports cancer metastasis [254]. In gastric cancer, activation of TGF-β1 in CAFs promotes mobility and invasion to lymphatic vessel in vivo [255]. In ovarian cancer, the activation of TGF-β2/SMAD upregulates the expression of CD44, MMP-9, and RHAMM via VCAN expression (encoding the chondroitin sulfate proteoglycan Versican) in CAFs to promote invasion and metastasis [256].

Additionally, transforming growth factor beta-induced protein (TGFBI, or βig-H3) is a matrix protein determined by TGFβ1 and secreted into ECM by various cancers [257]. Previous researches unveil that TGFBI stabilizes microtubules via FAK and Rho pathways [258]. Some researchers consider that TGFBI is a tumor suppressor because of the deficiency of TGFBI favors tumor formation [259], and high expression of TGFBI in tumor is associated with optimal chemotherapy sensitivity [260, 261]. However, TGFBI is a trouble maker to push cancer far away from primary location. In colorectal cancer, TGFBI promotes metastasis via stimulating β5 integrin/Src and disconnection of VE-cadherin junctions between epithelial cells [262]. In melanoma, TGFBI hinders cell adhesion to fibronectin, collagen-I, and laminin whereas co-localized with fibrillar fibronectin/TNC/periostin structures which favors invasive state of cancer [263]. In ovarian cancer, β3 integrin, against β1 integrin and syndecan-1, facilitates cell adhesion to TGFBI and induces migration and paclitaxel resistance [264]. Moreover, TGFBI sustains tumor cell survival after radiotherapy via stimulating FAK pathway activation in gastric cancer [265]. TGFBI also induces androgen deprivation therapy resistance, EMT, and bone metastasis in prostate cancer [266]. A research finds out that TGFBI causes decreased vessel perfusion and sever hypoxia, which favors metastasis in breast cancer [267]. Nevertheless, another research considers that TGFBI induce angiogenesis in colorectal cancer, indicating the organ-specific function of TGFBI in angiogenesis [268]. Intriguingly, TGFBI derived from non-cancer cells such as TAM in ovarian cancer also promotes cell invasion and metastasis [269, 270]. And the positive feedback loop of TGFBI secretion among TAMs/CAFs/TAMs constructs immunosuppressive TME [270], implying that the concentration of TGFBI in ECM is critical for metastasis but ignores its’ derivation (Fig. 3A).

By the way, ECM stiffness transduces mechanical signal to TWIST1, then destroys the interaction between TWIST1 and Ras-GTPase activating protein SH3 domain-binding protein 2 (G3BP2), which induces nuclear import of TWIST1 and EMT in tumor [271]. Mechanosensory receptor such as Ephrin type-A receptor 2 (EPHA2)/LYN protein also stimulates TWIST1 to induce EMT and invasion in responding ECM stiffness [272]. Other mechanosensors promoting EMT via several pathways such as transient receptor potential melastatin 7 (TRPM7)/SOX4 in breast cancer [273], CXCR4/ubiquitin domain containing 1 (UBTD1)/YAP in hepatoma [141] and EGFR in glioblastoma [274] have been reported. Attentionally, different tumor types rely on discrete EMT effectors [275]. A pan-genomic analysis links EMT phenotype with gene-coding proteins involved in the degradation of the ECM, supporting the oponin [276]. Because of different ECM program (generating acellular components in varying proportions) favors specific cancer progression, targeting characterized EMT biomarker is necessary.

### ECM-induced metabolism reprogramming: fueling the metastasis

Metabolic reprogramming facilitates the basic requirement for cancer cell survival and progression by providing energy and specific products to synthesize proteins [277]. Warburg effect, a phenomenon marked by overwhelming glucose uptake and a disproportionate utilization of glycolysis for energy production, could rapidly generates adenosine triphosphates (ATPs) and nicotinamide adenine dinucleotide phosphate (NADPH) to support malignant biosynthesis [278]. Aberrant metabolism pattern in cancer also correlated with ECM remodeling. Glycolytic enzyme uridine diphosphate (UDP)-glucose 6-dehydrogenase in cancer promotes the secretion of HA during the EMT process [279]. Targeting rate-limiting enzymes, such as phosphofructokinase-2/fructose-2, 6-bisphosphatase 3 (PFKFB3) of glycolysis is sufficient to suppress tumor migration and fibrosis [280, 281]. Additionally, ECM can shape metabolic reprogramming of cancer cells, thus enhance their aggressiveness. HA digestion stimulates glycolytic metabolism and glucose transporter protein type 1 (GLUT-1) translocation on the membrane of various cancer cell lines in vitro and in vivo [282]. To respond to HA degradation, zinc finger protein 36 (ZFP36) is rapidly upregulated and induces the degradation of thioredoxin-interacting protein (TXNIP), followed by enrichment of GLUT1 at the plasma membrane and enhanced tumor migration [282]. Signals of mechano-transduction from ECM stiffness to hepatoma triggers activation of MAPK/YAP pathway and accelerated aerobic glycolysis [283]. Intriguingly, increased collagen density sheds metabolic program of triple negative breast cancer (TNBC) cells from glycolysis to oxidative phosphorylation (OXPHOS), the metabolic pathway relies on mitochondria [284]. Another research investigates the impact of ECM stiffness on cancer cells in a 3D culture, demonstrating that softer ECM favors YAP pathway activation, glycolytic metabolism and proliferation [285]. By contrast, stiffer ECM induces upregulation of fibronectin 1 and MMP-9 expression, OXPHOS and lipid metabolism, and invasion [285]. Though some reviews focus on the crosstalk between ECM and metabolism [286], the complex relationship between ECM and tumor metabolism is still mysterious, further explorations are needed.

Though Warburg effect decreases the requirement for oxygen and relies on glycolysis, acid products such as lactate secreted into ECM may hamper tumor cell function. However, high concentration of lactate facilitates immunosuppression and sustains tumor progression via stimulating stromal cells [287]. For instance, collagen production in ECM dependents on CAFs, and the activation of pyruvate carboxylase (PC), a rate-limiting enzyme of aerobic glycolysis, of CAFs can be detected in TME [288]. Lactates fulfilling TME are ideal substrates of PC and sustaining non-essential amino acid biosynthesis [288]. Thus, secreted amino acids promote cancer progression in turn. Indeed, mechanical signal from ECM stiffness triggers glycolysis and glutamine metastasis in CAFs, then CAFs provide aspartate to support cancer proliferation and induce ECM remodeling [289]. Intriguingly, an acid concentration gradient from the center to the outside of the tumor tissue, which naturally forms the metastatic directionality, mediates cell mobility and invasion [290, 291]. Besides of CAFs, the crosstalk between mitochondria and ECM stiffness in cancer has been unveiled recently. Softer ECM of distant metastatic niche can enhance therapeutic resistance of cancer cells [292]. Precisely, cancer cells generate mitochondrial fission, which is the state that mitochondria are split into smaller components, and triggers the activation of nuclear factor E2-related factor 2 (NRF2) to enhance glutathione metabolism [292]. Therefore, the crosstalk between metabolism and mechanotransduction has called more attention recently and considered as a potential target for anti-cancer therapy (Fig. 3B).

When cancer cells are detached from ECM, increasing production of ROS is lethal for tumor cell survival [293]. Thus, elimination of ROS is critical for cancer metastasis. A systemic review has discussed metabolic mechanisms suppressing ROS generation, including the activation of pentose phosphate pathway (PPP), NRF2-induced catalase and superoxide dismutase 2 (SOD2), glutamine metabolism, and AMPK pathway-induced autophagy [294]. Warburg effect could stimulate PPP pathway and NADPH production, which suppress ROS generation.

Intriguingly, several researches of pro-metastatic role of glutamine in ECM remodeling have been reported. Physiologically, HIF-1α controls chondrocyte proliferation under hypoxia during endochondral ossification whereas prolonged HIF-1α stimulation replaces aerobic glycolysis by glutamine metabolism. During the process, chondrocytes alter their function from collagen production into collagen proline and lysine hydroxylation, which leads to skeletal dysplasia [295]. In cancer, the sustaining of proliferation of detached cells depends on AMPK/Nrf2-induced glutamine metabolism when detached from ECM [296]. Tumor cells also enhance their aggressiveness via several glutamine-associated mechanisms such as upregulation of glutamine receptor [297], tissue transglutaminase [298] and glutaminolysis [299]. Besides, cancer cell clustering triggers mitophagy activation, which can decrease the generation of ROS, to support tumor survival during metastasis [300]. The activation of mitophagy in cancer during the detachment from ECM depends on the expression of receptor-interacting protein kinase 1 (RIPK-1) [301], and secretion of ECM protein decorin [302, 303].

### Collagen orientation and CAFs: the fingerposts

Here, cancer cells are prepared for a long distant journey. The question is, where should they go? During normal development of organs, programs of cells have been well set from embryonic period to differentiation. Within a differentiated organ facing injury, cells just need to perform their duty to proliferation and migration which limited under a range of ECM [304]. The program will be shut off when everything becomes normal or cells meet their death. Nevertheless, cancer cells generating ECM create a gradient from primary loci of cancer to extravascluation site, then guide themselves’ migration in a certain direction. The organization of collagen, the most prevalent component in ECM, is critical for cancer progression [305, 306]. Clinical examinations have detected a profound association of collagen fiber orientation disorder to poor prognosis in breast cancer [307–309], Gastric cancer [310], and salivary gland cancer [311]. Pathological examination also detects prevalent disordered collagen orientation in various cancers [312–314]. A laboratory study demonstrates that high-density collagen co-culture transforms normal breast tissue into premalignancy [315]. Aged ECM transplantation also stimulates oncogenesis in breast cancer [316].

Importantly, collagen I-Matrigel composite extracellular matrix featured collagen orientation or less waveforms in ECM provides a contact guidance to typically induce directional migration of cancer cells [317, 318]. Several studies indicate that tumors prefer to invade and disseminate along radially aligned fibers rather than circumferentially oriented fibers [319, 320]. Moreover, collagen-rich environment mostly stimulates integrin signaling pathway and favors pre-metastasis niche [321]. In breast cancer, the balance between intracellular and cell–matrix adhesion determines ECM dynamics and the invasion of cancer cells [322]. Collagen I/α2β1 integrin triggers PI3K/Akt/SNAIL pathway to promotes metastasis in colorectal cancer [323]. In squamous cell carcinoma, ECM stiffness triggers collective invasion via mechanical sensing EGFR pathway-induced calcium absorption [324]. Additionally, when cancer cell invasion on aligned collagen fibers, the structure will lead cancer cell to invasion by sensing focal adhesion-mediated contact guidance [325]. In a 3D culture demonstrates that glycation enhances ECM stiffness and reduces directionality in aligned collagen gel, highlights the significance of posttranslational modification of collagen on tumor invasion [326]. These features together push cancer cells invasion, migration and metastasis in a certain direction.

CAFs are the primary derivation of acellular components including collagens in ECM [327]. Cancer stimulates CAFs proliferation and activation to facilitate ECM remodeling [328]. A ROCK-dependent paracrine axis has been recently identified and is responsible to breast cancer stimulated reprogramming of CAFs [329]. In PDAC, ECM stiffness induced by CAFs activates ERK pathway and promotes cancer metastasis [330]. In squamous cell carcinoma, IL-6 plays the same role by targeting STAT3/ERK pathway in CAFs [331]. Components derived from CAFs also provides bioactive signals for cancer progression. For instance, CAFs derived lumican promotes progression of gastric cancer via stimulating β1 integrin/FAK signaling pathway [228]. In breast cancer, CAFs expressing Hic-5 stimulates ECM remodeling and induces lung metastasis [332]. Matrix components laminin-5γ2 (LN-5γ2) triggers tumor budding, which defined as isolated single cancer cells or clusters of up to four cancer cells located at the invasive tumour front, of colorectal cancer cells [333, 334]. Mechanistically, LN-5γ2 interacts with β1 integrin to stimulate FAK and YAP activation in colorectal cancer [333], while increases density of stromal myofibroblasts in oral squamous cell carcinoma [335]. By contrast, decreased hyaluronan cross-linking mediated by CAFs favors invasion in breast cancer [336]. Attentionally, by using single cell high-throughput sequencing, specific CAF clones and their distinguished functions are precisely identified in PDAC [337, 338]. Intriguingly, a specific clone of CAFs associated with ECM (eCAFs) is identified in pancreatic cancer [339] and gastric cancer [340], and recently identified Endo180 and paired related homeobox 1 (Prrx1) may be significant genes associated with the function and plasticity of eCAFs [341, 342]. However, the precise role of eCAFs in cancer progression have yet to be elucidated.

Intriguingly, the orientation of fibers also contributes to the long-range distribution of CAFs [343]. Radially aligned ECM fibril constructed by cancer cells stimulates exosome secretion from CAFs to normal fibroblasts, therefore induces pro-oncogenic transformation of fibroblasts and tumor metastasis [344–346]. Moreover, activation of TWIST in CAFs at the tumor invasive front is associated with the expression of palladin and collagen α1, which facilitates arrangement of CAFs [347]. The concentration gradient of oxygen may also contribute to guide cancer cell metastasis. For instance, Hypoxia-induced Malic enzyme 1 expression and stimulates tumor budding, lactate production and YAP activation in oral squamous cell carcinoma [348]. Stress relaxion triggers procollagen-lysine, 2-oxoglutarate 5-dioxygenase 2 (PLOD2) expression and results in migration according to oxygen concentration [349]. In tumor tissue, hypoxia suppresses collagen I deposition, while a gradient deposition of collagen I may be associated to the oxygen supplement, which needs further studies (Fig. 3C) [350].

### Other factors point out the direction of metastasis

To adapt signals from ECM, changes on membrane-receptor in cancer cells are necessary to invade through the direction. DDRs are receptor tyrosine kinases that bind with collagen in an integrin-independent way [351, 352], and DDR1 can supplement the collagen-induced tumor progression when β1 integrin was eliminated [353]. Physiologically, DDR1 controls Mammary morphogenesis [354]. By contrast, DDRs play dual role to cancer cells. Downregulation of DDR1 triggers TGFBI secretion and tumor progression [355]. Collagen XV-mediated DDR1 suppression inhibits tumor invasion in PDAC [356, 357]. While activation of DDR1a/MMP2 promotes cell invasion in glioblastoma [358]. Collagen IV binds with DDR1 and mediates Src-dependent MMP2/9 secretion in TNBC invasion [359] and EMT in epithelial breast cell line [360]. Attentionally, the regulatory feedback between DDR1 and MMP secretion contributes to sustain the homeostasis in local ECM, highlights the significant role of DDR1 in ECM remodeling [361, 362]. For invasive directionality regulation, DDR1-induced Rho-GTPase member Cdc42 and its specific guanine nucleotide-exchange factor (GEF), Tuba is needed for ECM protein degradation [363]. Furthermore, inhibition of DDR2 induces decreased invasion of murine melanoma via ERK/NF-κB-mediated MMP secretion [364]. K14^+^ breast cancer cells expression DDR2 and CXCR4 and CAFs expressing DDR2 guides metastasis from primary tumor organoids to polarize to the leading edge and direct migration [365, 366]. Intriguingly, DDR2 mediates cell cycle arrest under the treatment of collagen, whereas deglycosylation of collagen can overcome the suppression in melanoma, implying the significance of collagen glycosylation [367].

Besides expression of ECM signaling receptors such as integrin and DDRs, expression of HA/CD44-mediated motility receptor RHAMM at the invasive front of colorectal cancer cells is necessary for invasion and metastasis [368]. It also mediates chemoresistance via activating TGF-β/Smad2 pathway [369]. Previous research demonstrates that HA/CD44 is essential for cancer cell bone metastasis [370]. After radiotherapy, a HA-rich environment constructed by activation of IL-1α/NF-κB/HAS2 pathway in cancer cells promotes glioblastoma metastasis [371]. These reports emphasize that the distribution of CAFs, together with cancer cells, is depends on the mechanical and bioactive signaling pathways (Fig. 3C).

Non-CAFs stromal cells also pave the way to invade ECM and guide cancer cells to metastasis. For instance, PSCs change the alignment of collagen fibers and induce ECM remodeling via Endo180 [372] and SPARC-dependent TGF-β/ROCK activation [129] to support cancer metastasis. In the omental environment of ovarian cancer, omental adipocytes, mesothelial cells and CAFs provides signals for cancer progression [373]. TAM polarization induces microtubules coherent within cancer cell and TAM and enhance tumor cell elongation [374]. Moreover, TAM-derived granulin stimulates hepatic stellate cells (HSCs) to secret periostin resulting in fibrotic microenvironment [375]. Bone-derived mesenchymal stromal cells (MSCs) secreted ECM components contribute to the metastasis [376]. The deficiency of stromal cell-secreted decorin stimulates colorectal cancer initiation and triggers ECM remodeling [377]. Other factor such as blood derived neural Wiskott-Aldrich syndrome protein (N-WASP) regulates lysophosphatidic acid recycling in a self-generated gradient and promotes PDAC metastasis [378]. Though a profound regulatory network within various cell types makes trouble to unveil precise therapeutic target, the program of ECM is the sally port for treatment designation, especially the identification of expression of ECM-associated enzymes.

### Matrix-related enzymes: crossing the red sea

LOX family, including LOX and LOXLs, plays a significant role in cancer-associated ECM stiffness by crosslinking extracellular matrix proteins, collagen and elastin [379]. LOX increases stiffness and stimulates FAK/Src pathway in colorectal cancer [380]. Hypoxia stimulates LOX expression and increase collagen crosslinking and tumor invasion in ovarian cancer [381]. Among LOX family, the profound association between LOXL2 and cancer progression has been reported. High expression of LOXL2 in CAFs is correlated with poor prognosis in colorectal cancer [382], prostate cancer [383]. And High expression of LOXL2 in tumor tissues predicts poor survival in cervical cancer [384], neck squamous cell carcinoma [385], hepatoma carcinoma [386] and other cancer patients [387].

Mechanistically, HIF-1α stimulates LOXL2 expression and tumor progression in hepatoma [388]. Additional LOXL2 treatment increases PI3K/Akt-dependent fibronectin deposition and lung metastasis in hepatoma [389]. A positive feedback loop between stiffness-induced zinc finger E-box binding homeobox 1 (ZEB1) expression and ZEB1/LOXL2-induced ECM stiffness remains tumor cells in a mesenchymal state [390]. Moreover, the competition between miR-200 and ZEB1 regulates collagen deposition and crosslinking, which stimulates β1 integrin/FAK/Src pathway, to promote invasion and metastasis [391]. Proinflammatory cytokines oncostatin M stimulates LOXL2 expression and collagen I crosslinking and invasion [390]. While overexpression of LOXL2 increases lung metastasis possibly via SNAIL1 upregulation in breast cancer [392]. Anti-LOXL2 treatment is sufficient to suppress tumor progression in breast cancer [393]. However, inhibition of LOXL2, which causes decreased matrix context and stiffness, promotes cancer progression in PDAC, suggests the organ specificity of ECM stiffness in oncogenesis [394]. Other LOX family proteins also participate in tumor progression whereas less results have been reported. For instance, LOXL1 supports intraductal xenograft of lobular breast carcinoma cells survival [395]. LOXL4 stimulates activation of programmed death ligand 1 (PD-L1), which induces immunosuppressive phenotype in macrophage, and facilitates an immunosuppressive microenvironment to promote hepatocarcinogenesis [396].

MMPs, the matrix-proteinases mediate generation of fragments from acellular components, and have long been associated with cancer invasion, metastasis and angiogenesis [397]. The role of MMPs in cancer progression and inhibitors of MMPs have been well reviewed recently [398]. Heat shock protein (HSP) family participates protein folding and maturation intracellular and associated with tumor progression [399]. Intriguingly, some researchers suggest that extracellular HSPs are critical to assist MMPs and promote tumor progression via ECM remodeling [400]. For instance, extracellular HSP70 and HSP90 are critical to interact with MMP2 and enhance migration in breast cancer [401]. The function of HSP90 and MMP2 interaction is under controlled by tissue inhibitors of metalloproteinase 2 (TIMP2) and ATPase homolog 1 (AHA1) co-chaperones [402]. AUY922, an inhibitor of HSP90, decreases fibronectin secretion into ECM and hampers invasion in prostate cancer [403]. Interestingly, inhibition of HSP90 facilitates decreased contractility and increased TGF-β2 expression of CAFs in prostate cancer [404]. Moreover, HSP47 regulates TGF-β to mediate ECM remodeling in glioblastoma [405]. In breast cancer, HSP47 interacts with non-muscle myosin IIA (NMIIAH) to promote contractile force of actin filaments [406]. HSP47 also promotes cell-platelet adhesion to mediate tumor invasion and metastasis via collagen I [407, 408]. Extracellular HSPs have demonstrate the potential to act as a therapeutic target for ECM-associated cancer treatment, whereas lacking enough evidences.

## ECM remodeling and angiogenesis in cancer

Unlimited expanding of cancer and increasing generation of ECM finally results in insufficient nutrients and oxygen, even impedes metastasis of cancer cells, theoretically. Angiogenesis is the process to form new blood vessels from pre-existing vessels, and it is essential to provide nutrients and oxygen for promoting tumor growth and hematogenous metastasis [409]. Besides, fibroblasts are critical in angiogenesis, while their function can be seriously controlled by cancer cells. A thorough review has concluded the role of CAFs and extracellular components generated from CAFs in tumor angiogenesis [410]. Briefly, ECM is essential for vascular integrity whereas sustaining pro-angiogenic signaling in tumors impairs the subsequent steps of vascular morphogenesis, namely the acquisition of a quiescent EC phenotype and the development of an intact and selectively permeable vascular barrier. In another word, in cancer, vascular basement membrane and ECM is destroyed and become less conjunctive with ECs. These features facilitate the advantage for cancer metastasis whereas enhance a potential permeability of immune cells, such as TAM (Fig. 3D) [410]. The influence of immune cells on ECM will be discussed latter.

Integrin signaling facilitates the basic stimulation of ECM-induced angiogenesis. For instance, β3 integrin induces VEGFR2 in ECs [411]. α6β1 integrin stimulated by VEGFA in ECs is essential for the formation of endothelial podosome rosettes, which promotes new vessel formation in tumor tissue, while laminin of the vessel basement membrane hampers the fucntion of α6β1 integrin [412]. Additionally, syndecan-1 is essential for new blood vessel maturation in cancer [413]. Chitinase-3-like protein 1 (CHI3L1/YKL-40), a glycoprotein secreted by various cancer cells and stromal cells, enhances the synergic effect of syndecan-1 and β3 integrin and activation of FAK/ERK pathway in ECs to promote angiogenesis in breast and colon cancer [414, 415]. Moreover, in glioblastoma, YKL-40 induces the synergic effect of syndecan-1 and β5 integrin and FAK/ERK-dependent VEGF secretion and VEGFR2 expression [416, 417]. In TNBC, β5 integrin facilitates cancer angiogenesis in vivo [418]. Syndecan-4 may also participate in the YKL-40-induced vessel formation [419]. Furthermore, tumor-derived ECM transforms normal endothelial cell and stimulate VEGF2 expression via β3 integrin/FAK/Src pathway in melanoma [420]. Low expression of β3 integrin in mural cells of blood vessel is associated with cancer progression [421]. Mechanically, losing β3 integrin in mural cells triggers phosphorylate FAK/HGFR/p65 and upregulation of CXCL1, C–C motif chemokine ligand 2 (CCL2) and TIMP-1, while CCL2 stimulates tumor progression via MAP/ERK kinase 1 (MEK1)-ERK1/2-ROCK2 pathway [421].

Recently, basement membrane multidomain heme peroxidase human peroxidasin 1 (hsPxd01) has been identified as a pivotal regulator to promotes angiogenesis via ERK, Akt and FAK pathway [422]. Peroxidasin crosslinks collagen IV and releases bromide to sustain ECs survival [423, 424]. Nevertheless, high expression of peroxidasin could be detected in invasive melanoma cells rather than non-invasive cells and generates hypobromous acid, suggests the additional reaction with other biomolecules which have yet to be elucidated [425]. ECM component elastin microfibrillar interface protein 2 (EMILIN2) binds on EGFR and stimulates angiogenesis, mediates sensitivity to chemotherapy [426]. The absence of Multimerin-2 (MMRN2), a member of EMILIN family expressed by ECs, hampers VEGFA/VEGFR2 activation and angiogenesis in xenograft model [427, 428]. The expression of matrix protein CLEC14A combines with MMRN2 and reactivate angiogenesis in cancer [429, 430]. MMP-9 mediates MMRN2 degradation and enhances tumor angiogenesis [431]. Intriguingly, blocking CD93-MMRN2 interaction in ECs favors angiogenesis and metastasis in tumor [432]. MMRN2 combines with and stabilizes CD93, therefore stimulates downstream pathways including β1 integrin, FAK and fibronectin expression [433]. Thus, MMRN2 is a potential biomarker for anti-angiogenesis therapy.

## ECM remodeling and lymphangiogenesis in cancer

The lymphatic system is indispensable for the collection and cycling of tissue-extravasated fluids, macromolecules and immune cells into the bloodstream, especially for tumor metastasis through lymphatic vessel [434]. The canonical pathway for lymphangiogenesis in cancer is the combination of VEGF-C/D and VEGFR-3. The interaction would trigger activation of downstream pathways including Akt phosphorylation [435]. Factors and signaling pathways associated with lymphatic formation have been introduced whereas ECM role in lymphangiogenesis is unclear [436].

During wounding healing and inflammation, a fibrin-binding variant of VEGF-C induces lymphangiogenesis and ECM deposition [437]. ECM stiffness stimulates GATA binding protein 2 (GATA2) and GATA2-dependent VEGFR-3 expression, which mediates lymphatic endothelial cell growth and migration in vivo [438]. HA increases expression of VEGF-C and VEGF-D in tumor-stromal interfaces to mediate lymphangiogenesis [439, 440]. Soluble factors such as heparanase stimulates VEGF-C expression in cancer [441, 442], while TGFBI induces activation of FAK signaling pathway in lymphatic endothelial cells (LECs), and increased expression of CCL21 on the surface of LECs to induce the dissociation of VE-cadherin junctions between LECs [443, 444]. Additionally, expression of membrane glycoprotein podoplanin increases lymphatic vessel formation in oral squamous cell carcinoma [445]. Podoplanin also expressed on the membrane surface of TAM, links TAM and LECs to mediate ECM deposition and lymphangiogenesis via β1 integrin activation in breast cancer [446].

Another potential target associated with malignant lymphatic formation is a collagen-associated binding protein: collagen- and calcium-binding EGF domains 1 (CCBE1), an indispensable regulator for embryonic lymphangiogenesis [447]. Physiologically, CCBE1 promotes VEGF-C expression in a posttranslational layer to transform inactive form of VEGF-C into a mature form [448]. Further research unveils that the activation of CCBE1-induced VEGF-C is depend on the EGF domain of CCBE1, whereas collagen domain is essential for CCBE1 activation [449]. Precise mechanism of CCBE1-induced VEGF-C activation is the enhancing of cleavage activity of a disintegrin and metalloproteinase with thrombospondin motifs 3 (ADAMTS3) and the facilitating of the colocalization of VEGF-C and ADAMTS3 [450]. Indeed, both cancer- and CAF-secreted CCBE1 mediates activation of VEGF-C within ECM and induce lymphangiogenesis and tumor progression [451]. Expression of TGF-β in cancer cells and CAFs transcriptionally suppresses CCEB1 expression by activating downstream SMAD [451]. Besides, high expression of CCBE1 is associated with tumor progression and drug resistance in gastrointestinal stromal tumor [452] and colorectal cancer [453]. Suppression of CCBE1 by miR-330-3p hampers breast cancer metastasis [454]. However, the downregulation of CCBE1 expression could be detected in ovarian cancer and associated with metastasis, implying a potential organ specific role of CCBE1 (Fig. 3D) [455].

Generally, tumor lymphatic metastasis can benefit from enhanced lymphatic vessel formation, whereas the process can also provide more chances for immunocytes to migrate through the lymphatic vessel. Nevertheless, immunosuppressive environment commonly identified to be accompanied with abnormal ECM remodeling, which hampers the function of immune cells to recognize and eliminate cancer cells. Thus, it is crucial to uncover the precise mechanism of ECM-mediated immunosuppressive microenvironment.

## ECM remodeling and immunosuppression in cancer

A pro-oncogenic ECM featured by collagen crosslink and glycoprotein-presented bioactivators transduce signals to regulate functions of immune cells. These factors not only mediate cytoplasmic signaling to promote tumor progression and suppress immunoreaction, an impenetrable shield constructed by remodeled ECM also hampers immuno-infiltration. Collagen density and tissue stiffness is pivotal for the infiltration of immunocytes. For instance, a 3D culture constituted by various density of collagen unveils the changes in for T cell composition, while high collagen favors high ratio of CD4/CD8 T cells and lower activity of CD8 T cells [456]. Different colonies of infiltrating T lymphocytes are changed in number, surface markers, subsets and gene expression under the stress from ECM stiffness [457]. Additionally, tumor-modified arrangement of collagen, which also known as collagen orientation, hinders the infiltration of immunocytes. A real-time microscopy motoring the trace of CD8 T in ovarian tumor tissue demonstrates that the mechano-gradient formed by peritumoral collagens guides CD8 T cells moving lingering in a certain direction whereas waken their infiltration to intrude the tumor islets [458]. Therefore, similar to cancer cells, previous discoveries suggest that migration of immune cells in ECM depends on the distribution and interaction among ECM components, especially among fibril collagen network [459]. Additionally, the influence of ECM stiffness on other immune cells such as dendric cells maturation [460] and macrophages [461, 462] have been recently reported. These remodeled ECM composite a maze, with impenetrable wall and winding roads, makes immune cells lost their way. To precisely explore the variation in immune cells surrounded by ECM is essential for further understanding of immune-oncology. Here, the relationship between ECM stiffness or ECM components with immune cells will be discussed.

### ECM remodeling and immune cell migration

**Fig. 4:**
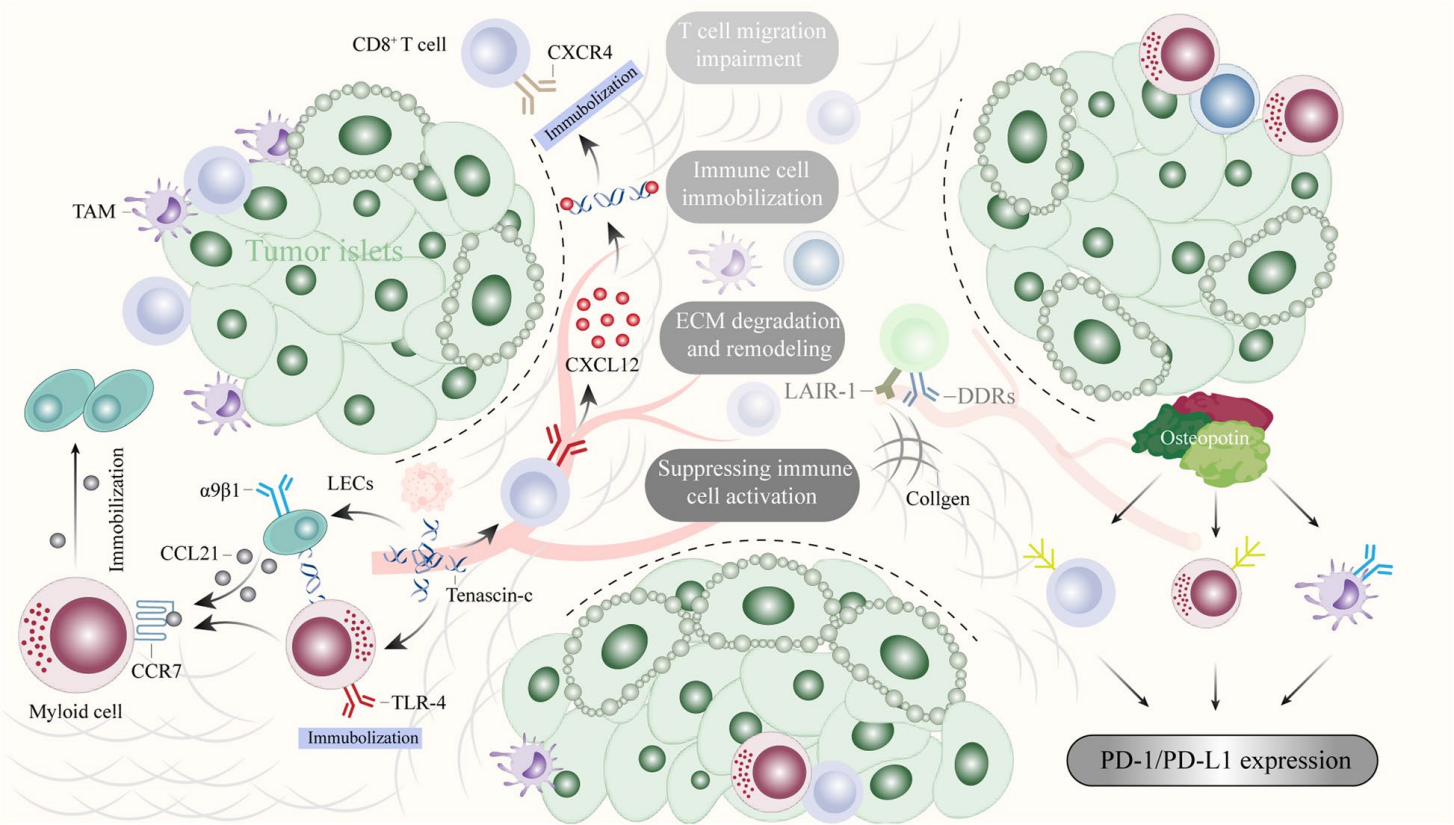
ECM hampers activation and migration of immune cells. ECM constructed immunosuppressive environment demonstrate various mechanisms to induce immune escape in TME. Firstly, increasing density and modification of ECM components strikingly enhance the stiffness, then hamper immune cell migration into tumor islets by composing a physical barrier or directly cell-ECM surface inhibition (via interaction or receptors such as DDRs and LAIR-1). Moreover, tenascin-C immobilizes T cells with immunosuppressive cells and stromal cell, thus immune cells are fixed in the ECM. Finally, OPN stimulates PD-1/PD-L1 expression in immune cells, thus induce immunosuppression

Collagen crosslink and orientation constructs a stiff and parallel fibril alignment around tumor cells. It’s need to be emphasized that the architecture of collagen fibril shows less impact on immune cell infiltration within tumor tissue, but hamper immune cell migration into the inside, or the islets in another word, of tumor. The phenomenon could be observed in several results [313, 463, 464]. A possible hypothesis is that the proliferation of tumor cell is unlimited, matrix stiffness derived from collagen crosslink contributes to retain the pressure inside the tumor tissue and constructs a pressure gradient, which push not only immune cell but also agents away from cancer cells. While at least, new vascular and lymphatic vessels induced by tumor would not be collapsed by interstitial fluid pressure, but by ECM remodeling [465]. Thus, matrix stiffness conducts both mechanical and contact signaling to mediate immune cell migration (Fig. 4).

In ovarian cancer, ECM stiffness hampers migration of T cell from peritumoral stroma into tumor islets, while degradation of matrix overcomes the situation [313]. This research, together with a discovery from PDAC, which demonstrates that expression of T-cell-active chemokines and β-integrin pathway is irresponsible to the intertumoral T cell-infiltration, highlights the pro-migration role of collagen density on T lymphocytes [466]. Moreover, matrix stiffness increases the CD4/CD8 T cell ratio, and decreases their activation [456, 467]. Mechanistically, CD4 T cell can form a mechanically complex with stiff matrix surface, this interaction modulates T cell cytoskeletal organization which may suppress T cell activation [468]. Another research using hydrogel-integrated culture unveils that stiffer ECM triggers IL-2 secretion whereas reduced proliferation of Jurket T cell (a human T lymphocyte cell line) [469]. Therefore, ECM remodeled by tumor shows immunosuppressive function on T lymphocytes, not only by suppressing their activation, but also by changing their composition.

For antigen-presenting cells, such as macrophages and dendritic cells (DCs), ECM stiffness acts as a disparate role. Increasing stiffness of ECM enhances M2 polarization and HIF-1α-induced LOXL2 expression via β5 integrin/FAK/MEK/ERK pathway of macrophage in hepatoma [470]. Intriguingly, soft matrix physiologically induces mitochondrial ROS generation in a Rho-GTPase-depend way and M1 polarization in macrophage [471]. These results suggest that matrix stiffness may enhance the activation and M2-like polarization of macrophage, which tends to support tumor progression. For DCs, matrix stiffness enhances expression of C-type lectin, which mediates antigen internalization [472]. Stiff matrix stimulates glycolytic metabolism and YAP/TAZ pathway to enhance proliferation and activation of DC [460]. By the way, HA, another widely expressed protein to construct ECM stiffness, also transforms macrophages into M2-like polarization via targeting CD44, and induces apoptosis of neutrophil even activation of DCs via binding with TLR-4 [473]. Thus, unlike T cell, antigen-presenting cells respond positively to ECM stiffness, whereas the function is advantageous for tumor progression.

Additionally, though less research targets the role of ECM stiffness in regulating Treg, some clues are worthy of reference. For example, activation of α4β1 integrin, an ECM-associated receptor, enhances immunosuppressive function of Treg [474]. High expression of leukocyte-associated Ig-like receptor 2 (LAIR-2), an inhibitor of immune inhibition receptor LAIR-1, in Treg is correlated with poor outcome in lung adenocarcinoma [475]. A significant observation demonstrates that losing of hyaluronan and proteoglycan link protein 1 (HAPLN1), a hyaluronic and proteoglycan link protein, in skin could trigger collagen alignment in melanoma, which hampers CD8 T cell migration whereas enhances infiltration of myeloid-derived suppressor cells (MDSCs) and Treg in melanoma [476]. Besides, Treg influence local immunoreaction via interaction with CAFs. In breast cancer, CAFs secrets IL-6 and enhances adenosine generation from CD73 + γδT cells by stimulating STAT3 activation. Then, adenosine stimulates IL-6 secretion by CAFs via adenosine/ adenosine A2 receptor (A2BR)/p38MAPK pathway and constructs a positive feedback loop between Treg and CAFs [477]. A single-cell RNA sequencing about the heterogeneity of fibroblasts in TNBC also indicates the significant correlation between Treg and CAFs [478]. It’s worth to further explore the relationship between Treg and CAFs in various cancers.

However, in PDAC, collagen seems as a trouble maker for cancer survival. The breakthrough of stromal cells role in pancreatic cancer immunosuppression is the identification of different subtypes of CAFs, which preliminarily classified into αSMA-expressing myCAFs, associated with desmoplastic stroma generation, and IL-6/leukemia inhibitory factor (LIF)-expressing inflammatory fibroblasts (iCAFs), associated with inflammation [337]. myCAFs located adjacent to tumor cells whereas iCAFs located more distantly. After that, MHC class II-expressing antigen presenting CAFs (apCAFs) have been identified, and their role in stimulating activation of CD4 T cells via antigen-specific manner has been uncovered [338]. Further investigation finds out that IL-1 and TGF-β oppositely regulate IL-1R/JAK/STAT activation and induce differentiation of CAFs into iCAFs (IL-1), or myCAFs (TGF-β) [32]. Importantly, when generation of collagen I from spinal muscular atrophy (SMA)^+^ CAFs is reduced, spontaneous PDAC formation mice incline to meet poor survival and immunosuppressive environment [33]. Collagen I depletion in tumor tissue results in enhanced oncogenesis and increased expression of Cxcl5, which causes recruitment of myeloid-derived suppressor cells and suppression of CD8 + T cells in a CXCR2/CCR2-dependent manner [33]. Further investigation using 3D matrices and time-lapse microscopy unveils the distribution of T cell within pancreatic tumor tissue doesn’t correlate to the orientation of collagen alignment [479]. These results depict a distinguish role of collagen in PDAC compared to other cancers.

### DDRs and LAIRs: collagens’ best friends

As previously discussed, DDRs are critical receptors sensing collagen and mediates tumor progression. Expression of DDRs in immune cells is also pivotal for cell migration and cell-ECM adhesion. Under the stimulation of collagen, in renal cancer, high expression of DDR1a in monocytes mediates infiltration and migration in ECM [480], while DDR1b triggers MAPK/NF-κB pathway to activate macrophage during inflammation [481]. In a 3D collagen culture, DDR2 expression in neutrophils induces MMP secretion and local generation of collagen-derived chemotactic peptide gradients to regulates directionality [482]. In addition, suppressing DDRs in cancer contributes to the immunoactivity. A pan-cancer in vivo model verifies the efficiency of suppression on DDR2, which significantly enhances the efficacy of anti-PD-1 immunotherapy [483]. Recently, a novel discovery unveils that DDR1 extracellular domain (DDR1-ECD) mechanically binds with collagen and mediates collagen fiber alignment, which hampers T cell infiltration, suggests as a potent therapeutic target for immunotherapy [484].

LAIR-1 (or CD305) is an immune inhibitory receptor prevalently expressed by almost all types of immune cells and its’ activation triggers immunosuppression. In chronic lymphocytic leukemia, oral squamous cell carcinoma, breast cancer and hepatoma, expression of LAIR-1 is negatively correlated with severity of illness [485–489]. In ovarian cancer, CD11c^+^CD11b^−^CD103^+^ DCs could be observed in tumor tissues, while PD-1 expression is positively correlated with LAIR-1^high^ in DC [490]. Collagen is the ligand of LAIR-1 in ECM. In melanoma, collagen mediates CD8 + T cell exhaustion through LAIR1/Src homology 2 domain-containing protein tyrosine phosphatase 1 (SHP1) pathway while combination of programmed death 1 (PD-1) blockades with overexpression of LAIR2, a soluble homologue of LAIR-1 with higher affinity for collagen and thereby acts as a decoy receptor, significantly overcome tumor growth and lung metastasis [491, 492]. MMP1/9-mediated collagen I fragment production targets LAIR-1 to suppress CD3 pathway and interferon gamma (IFN-γ) secretion in T cells [493]. A LAIR-2-Fc recombinant protein also suppresses tumor progression and re-activate anti-tumor immune response [491, 493, 494]. Especially, double blockage of LAIR-1 and TGF-β can support totally tumor elimination by PD-L1 [495]. However, A research reports that overexpression of LAIR-1 suppresses ovarian tumor growth via inhibiting PI3K/Akt/mTOR pathway [496]. While the in vivo experiments of this research are based on nude mice, which lacks specific immunity. Thus, LAIR-1 primarily acts as an immunosuppressor rather than a tumor inhibitor.

### TNC and Osteopotin: immune cells shall not pass!

Proteins secreted by cancer cells and stroma cells are seductive traps to immune cells. Some are neighborly while some are vicious. The role of collagen [497] and other ECM components [498] in immunoreaction in cancer has been well reviewed. Among them, TNC and osteopotin act as the rising stars for immunotherapy.

#### TNC

Overexpression of TNC could be specifically detected and associated with immunosuppression and sever progression in various cancers, such as NSCLC [499], breast cancer [500] and oral tongue squamous cell carcinoma [501]. ECM from glioblastoma cell line culture expressing TNC shows suppressive function on Jurkat T cell migration [502]. During the formation of PDAC in vivo model with normal immune function, the existence of TNC promotes oncogenesis and activation of Wnt singling pathway in cancer cells [503]. Further investigation finds out that TNC interacts with α5β1 integrin and suppresses cytoskeleton reorganization of T cells, then supports the survival of lymph node metastatic prostate cancer [504]. In glioblastoma, cancer cells secrets TNC-expressing exosome, then TNC targets α5β1 and αvβ6 integrins to suppresses mTOR singling and activation of T cells [505].

Intriguingly, another research suggests that CD47, which mediates immune evasion via hampering phagocytosis, negatively controls TNC secretion in glioblastoma cells [506]. While the increasing expression of TNC enhances tumor necrosis factor alpha (TNFα) secretion from TAMs via TLR-4/STAT3 pathway [506]. The similar function on TLR-4 could be observed in microglial, the specific type of macrophage in neuro system [507]. It seems like TNC enhances immunoactivities of macrophage. However, more studies demonstrate that TNC triggers immunosuppression via regulating macrophage in the TME. 

Breast cancer-derived TNC transforms TAMs into M2-like polarization and triggers immune-suppressive phenotype whereas inhibition of TLR-4 enhances PD-L1 immunotherapy [508]. In oral squamous cell carcinoma, TNC/TLR-4 increases the expression of CCR7 in CD11c^+^ myeloid cells [509]. Meanwhile, TNC/α9β1 integrin induces CCL21 expression and binds on CCL21 in LECs [509]. Thus, CD11c + myeloid cells and LECs are co-located and hijacked by TNC and construct a lymphoid immune-suppressive stromal environment [509]. The similar function of the anchoring effect of TNC has been reported recently. In TNBC, TNC/TLR-4 induces CXCL12 expression in CD8 T cells and retains CD8 T cells within the ECM and decreases immunoreaction via CXCR4/CXCL12 (Fig. 4) [510]. The two reports hightlight the novel role of TNC in limiting immune cell within peritumoral stoma, and the function further hampers immune cell migration and provides a immunosuppressive environment. For the upstream factors of TNC, JNK role in stimulating TNC expression has been discussed. In addition, TNBC with autophagy-deficiency could ubiquitinate TNC and sustain TNC expression by SKP2, therefore induce immunosuppression [511]. TNC may also combine with secreted TGF-β and stimulate activation of TGF-β, whereas the precise mechanism is still unclear [250].

#### Osteopotin (OPN, or SPP-1)

Previous research has unveiled that OPN is the ligand to CD44 and integrin, and contributes to the anoikis resistance and promoting adhesion in ECM [512]. Some reports targeting the role of intracellular OPN in immune regulation. For instance, expression of intracellular OPN is controlled by T-bet signaling pathway and regulates migration of T cells [513], as well as induces IFN-α expression via TLR-9 in plasmacytoid DCs [514]. In macrophage, intracellular OPN interacts with MyD88 to suppress TLR singling pathway, therefore inhibits inflammation-stimulated formation of hepatoma [515]. Further investigation demonstrates that both intracellular and secreted isoforms of OPN are dispensable to stimulate activation of DCs [516]. Intriguingly, DCs under hypoxia, which is commonly detected in TME, shows increasing secretion of OPN and impaired immune activation to support tumor immune escape [517]. Another research in a spontaneous breast metastasis model suggests that secreted OPN is mostly derived from cancer cells and supports tumor survival during metastasis, while high expression of intracellular OPN in MDSCs induces immunosuppression around metastatic site [518].

Indeed, additional investigations highlight the role of secreted OPN in immunosuppression. Secreted OPN form tumor cells contributes to the accumulation of MDSCs via targeting CD44 and stimulating downstream ERK/MAPK pathway to induce extramedullary myelopoiesis and immune escape [519]. Similar result could be detected in *helicobacter pylori*-induced gastric cancer, another inflammation-associated cance [520]. Precisely, high expression of intracellular OPN results in increased expression of PD-1 in macrophage and lung cancer cells via NK-κB pathway [521, 522]. Whereas in glioblastoma, tumor secreted OPN targets αvβ5 integrin to recruit and induce M2-like polarization of macrophage [523]. Interferon regulatory factor 8 (IRF8) transcriptionally suppress secreted OPN expression in CD11b^+^Ly6C^low^Ly6G^+^ myeloid cells, which caused decreased interaction between OPN and CD44 receptor in T cell and suppresses immune escape in colon cancer [524]. High expression of secreted OPN in hepatoma stimulates colony-stimulating factor 1 (CSF1)/CSF1R activation in macrophage which further increases PD-1 expression in hepatoma cells [525]. While in PDAC, WD repeat domain 5 (WDR5), a histone methyltransferase, mediates H3K4me3 on promoters of OPN and CD44 and enhances their expression to promote PD-L1 expression in tumor cells and MDSCs [526]. Thus, OPN links expression of PD-1/PD-L1 expression in both cancer cells and immune cells to induce immunosuppression.

By the way, specific transcripts from secreted phosphoprotein 1 (SPP-1) are critical for distinguished function of OPN. Overexpressed three different transcripts of OPN in breast cancer unveils similar oncogenic role but different immunosuppressive role in three trascripts [527]. Importantly, orally OPN administration suppresses tumor growth in mice with normal immune function, highlights the significance of endogenous OPN in promoting tumor progression [528]. Indeed, some results have proven the hypothesis. The isoform of secreted OPN from glioblastoma could not be observed from normal human astrocytes [529]. This isoform triggers macrophage M2-like polarization and tumor progression [529]. Another research suggests that the specific fragment of OPN cleaved by MMP-9 is sufficient to trigger MDSC infiltration and immune suppression [530]. The expression of membrane-anchored protein a disintegrin and metalloproteinase domain 8 (ADAM8) in glioblastoma cells and macrophages mediates OPN secretion and suppresses angiogenesis via JAK/STAT3 pathway, may also contributes to the formation of the specific isoform of OPN in ECM [531]. Thus, antibody targeting specific OPN in cancer could be an optimal option [532].

### TAM-induced ECM remodeling: the betrayer

TAMs are accompanied with tumor cells to composite pro-oncogenic environment. Under the stimulation from cancer cells or ECM environment, TAMs mediate matrix remodeling and contributes to the migration of cancer cells in a certain direction. TAM shapes ECM remodeling by high-rate degradation of the matrix and generation of ECM proteins [533]. In colorectal cancer, depletion of TAM results in a huge variation of expression of ECM components. ECM fragments from TAM-sufficient tumors triggers tumorigenesis compared with TAM-deficient tumors [73]. Deficiency of TAM strikingly decreases density and crosslink of collagen, especially reduces collagen I and XIV expression in CAFs [73].

Additionally, the secretion of MMPs from TAMs acts as a pivotal role in carving out cancer cells’ way to metastasis. High expression of MMP-11 in TAMs is associated with poor outcomes in breast cancer patients [534]. Overexpression of MMP-11 in macrophages, but not in cancer cells, increase monocyte recruitment and migration of Her-2^+^ breast cancer cells via CCL2/CCR2/MAPK pathway [534]. Furthermore, aberrant expression of genes in TAMs controls ECM remodeling to provide the advantage for tumor migration. Overexpression of B7-H3 in TAM shows fewer collagen fibers and enhanced angiogenesis in breast cancer [71]. In TNBC, high podoplanin expressing TAMs has been identified to adhere to LECs [446]. The combination of glycosylated podoplanin from TAMs with galectin 8 on LECs stimulates the activation of β1 integrin. This function facilitates angiogenesis and lymphangiogenesis via localized ECM remodeling [446]. Disabled homolog 2 mitogen-responsive phosphoprotein (DAB2) positive TAMs, which localized at the tumor-invasive front, promotes metastasis via matrix remodeling [535]. Precisely, knockdown of DAB2 impairs integrins’ surface distribution and their ability to internalize ECM fragments, under the expression of α5β1 integrin and stiffer ECM [535]. By contrast, ECM stiffness stimulates DAB expression via mechanically upregulating YAP/TAZ pathway, triggers ECM remodeling and integrin-dependent migration, which assists tumor invasion [535]. Therefore, TAMs act as tour guides, pointing out the way out for them from primary tumor tissue to distant metastatic sites.

Other immune cells also participate to the formation of ECM remodeling. Increasing stimulation from tobacco will sustain a lung inflammation, neutrophils response to the inflammation and secrets neutrophil elastase and MMP-9 [536]. Unfortunately, laminin would be cleaved by MMPs and remodeled laminin then awakens quiescent cancer cells via stimulating α3β1 integrin signaling pathway [536]. Besides, BMDC derived ECM miR-92a induces activation of HSCs, subsequently increasing ECM deposition [537]. Roles of other immune cells, such as NKp46-expressing NK cells [538]**,** LOX-expressing T cells [539] and TGF-β/CD73/adenosine-dependent Myeloid cells [540] in ECM remodeling and tumor progression are recently reported. After all, the profound regulatory network between ECM and immune system is the treasure, which provides targets to us to validate their potential in anti-tumor treatment.

## Anti-cancer therapy targeting ECM

Raising morbidity of cancer worldwide demonstrates the emergency of precise treatment strategy design and the discovery of novel therapeutic targets [541]. Canonical therapeutic strategies including chemotherapy, radiotherapy, as well as biomarker-based therapies such as endocrinotherapy and targeted therapy significantly increase cancer patients’ prognosis. Nevertheless, aberrant gene expression and disordered signaling pathway of tumor cells shape pro-tumorigenic ECM remodeling, which forms the tough environment to impede infiltration of anti-tumor immune cells and agents, as well as sustaining cancer cell survival under the stress from anti-tumor agents [542].

**Fig. 5:**
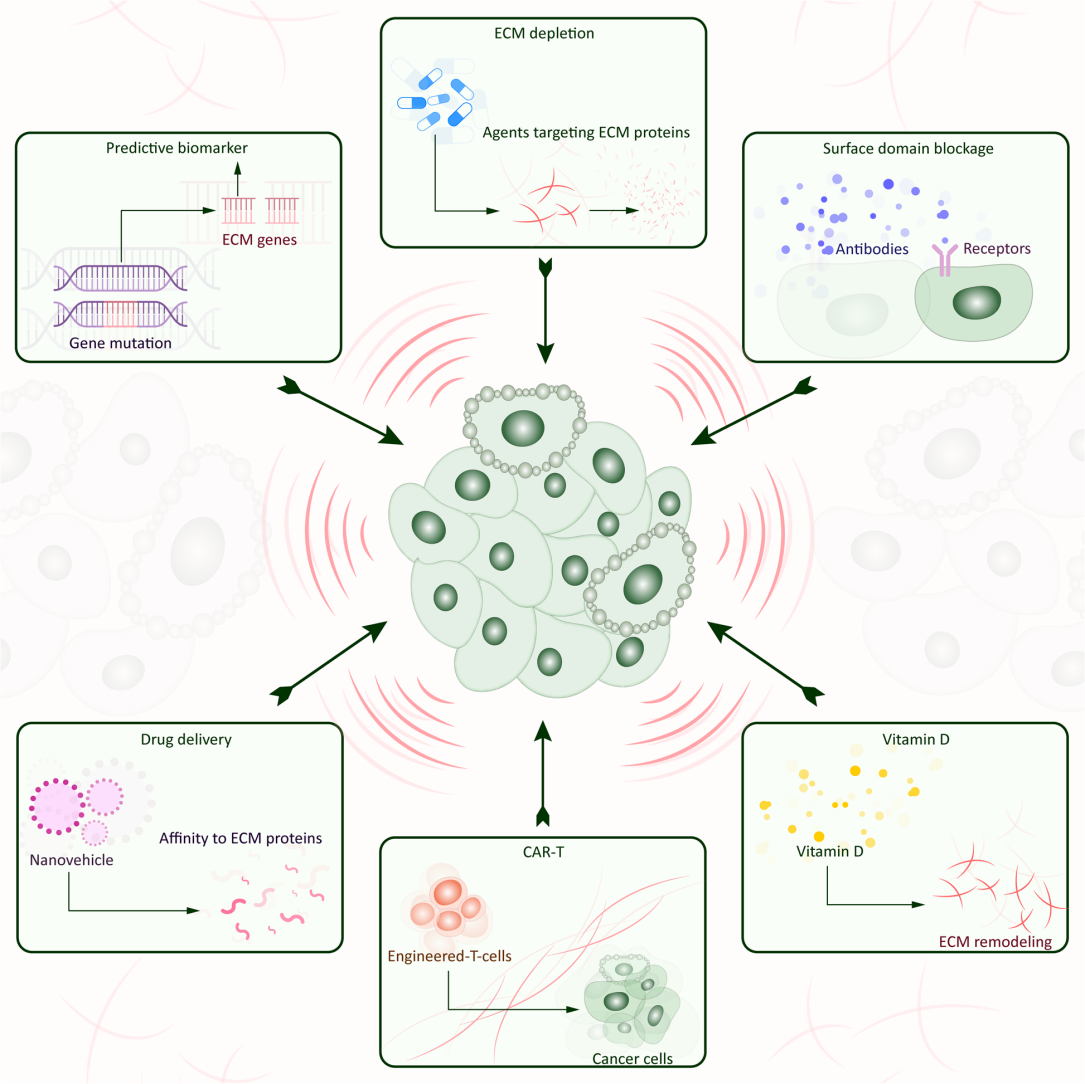
The diagram of ECM-targeting treatment. Several strategies targeting ECM could be optimal selections for anti-cancer therapy. However, these strategies are far from the clinical application. Further investigation is needed to verify the therapeutic effect of these strategies

ECM shaped by cancer cells links multiple drug resistance. Hydrogel constructed matrix mimics ECM stiffness and unveils enhanced chemoresistance in breast cancer [543, 544], and glioblastoma [545]. A reasonable model constructed by breast cancer patient derived scaffold which perfectly mimic acellular structure of ECM and unveiled enhanced drug resistance in cultured cancer cells [546]. Cancer cells 3D culture of HA also shows severe chemoresistance, especially for glioblastoma cells [547, 548]. On the other hand, chemotherapy can push remodeling of acellular matrix components, stiffness and matrix adhesion, which results in chemoresistance. For instance, after chemotherapy treatment, a positive feedback loop between THBS2-deficient CD133^+^ liver CSCs and local soft ECM induces cell metastasis in hepatoma [549]. In ovarian high-grade serous carcinoma, cells with high upregulated expression of collagen VI strikingly stiffen ECM and induce chemoresistance via enhancing integrin-based cancer cell adhesion on stiff collagen VI substrate after cisplatin-based chemotherapy [550]. Intriguingly, soluble laminin-322 secreted by HSCs in conditional medium of hepatoma can induce sorafenib resistance by sustaining activation of α3 integrin/FAK pathway, implying is that the components of ECM that matter, not their location [551]. Another research finds out that a short-time contact between placenta ECM and Erα^+^ breast cancer cells stimulate α5 integrin-dependent autophagy and paclitaxel resistance in cancer cells, highlights the critical role of ECM in chemoresistance [552]. Thus, targeting ECM is an optimal method assisting chemotherapy, even enhancing the efficacy of other therapies, and several strategies will be discussed here (Fig. 5).

### ECM signature as predictive biomarker

Each tumor tissue featured by unique gene signature contains particular ECM composition. ECM gene signature may help to identify the sensitivity of cancer to therapy. Several attempts have been made by previous studies. For example, specific stromal-related gene signature is correlated with prognosis of large-B-cell lymphoma patients receiving chemotherapy [553]. Similar association between ECM gene signature and outcomes of patients could be observed in other cancer [554–557]. Besides gene signature, proteomic analysis of ECM proteins in cancer also contributes to identify cancer and normal tissues [558]. Recently, a study demonstrates that matrix components released in blood after anti-PD-1 treatment in melanoma is associated with prognosis of patients [559]. Among them, collagen III and vimentin contribute to the prediction of the efficacy of PD-1 inhibitor [559].

Multiple predictive signatures may provide a method to analyze patient outcomes. While the designation of treatment based on these results is difficult to decide. Fortunately, ECM gene expression is commonly correlated with genes that has been well studied. Detection of these genes would contribute to the designation of treatment. For instance, ECM signature genes are identified under the control of TGF-β1, and expression of COL11A1, one of the signature genes, is upregulated during ovarian cancer progression [560]. Moreover, B-Raf proto-oncogene (BRAF) mutation (BRAF^V600^) in melanoma favors collagen-rich environment generated by CAFs, while dual targeting DDR1 and DDR2 contributes to the efficacy of imatinib in vivo [561]. Thus, identification of pivotal gene in ECM remodeling is critical for therapeutic designation.

### ECM degradation

A reasonable treatment to increase migration of anti-tumor immune cells and agents is normalizing ECM remodeling. Matrix-associated enzymes, including MMPs, LOX and LOXLs, are primary factors modify ECM structure. Targeting these enzymes to mediate mechanical signals and enhance the efficacy of immunotherapy in cancer shows great potential [562]. Though suppressing of MMPs could suppress cancer cell migration and invasion, it also hampers infiltration of other cells and agents. Thus, other factors inducing ECM degradation such as nitric oxide (NO) [563] and HSP70 [564] are potential targets. A recent study indicates that even through an intertumoral injection, ECM stiffness strikingly hampers drug delivery compared with soft one [565]. Thus, LOX and LOXs, which mediates collagen crosslink in tumor cell, are optimal targets to alleviate ECM stiffness. Suppression of LOXL2 is effective for tumor suppression. Beta-aminopropionitrile (BAPN) is the pan LOX inhibitor. Several research has proved that BANP treatment can suppress proliferation and invasion of tumor cells [395, 566, 567]. In osteosarcoma, a positive feedback loop between c-Fos/AP-1/Wnt pathway and LOXL2-induced matrix crosslink, while BAPN destroys the feedback loop [568]. A monoclonal antibody (AB0023) targeting LOXL2 inhibits stoma generation and TGF-β activation in cancer and shows more efficacy and safety than BANP [569]. Moreover, aminomethylenethiophenes (AMTs) is a potent oral bioavailable anti-tumor agent targeting dual LOX and LOXL2 and exhibit improved pharmacokinetic properties and excellent antitumor efficacy in vivo [570].

Besides, HA is one of the most prevalent ECM components and contributes to the formation and progression of cancer. 4-methylumbelliferone (4-MU), the HA synthesis inhibitor, significantly decreases HA expression and tumor progression in ER-negative breast cancer [571] and hepatoma [572], it also overcomes chemoresistance in ovarian cancer [573]. Oral dietary supplement of 4-MU is effective to suppress prostate cancer progression via abrogating HA signaling in vivo [574]. Intriguingly, targeting metabolic metabolism associated with HA synthesis may be another selection. A small molecule glutamine analog targeting glutamine-fructose amidotransferase 1 (GFAT1), the rate-limiting enzyme of HA synthesis, decreases renewal and metastatic ability of tumor cell, and increases sensitivity to anti-PD-1 treatment [575]. The combination of 4-MU and anti-metabolic therapy (dichloroacetate, the pyruvate dehydrogenase kinase inhibitor) strikingly suppresses esophageal cancer progression in vitro and in vivo [576]. Moreover, the combination treatment of 4-MU and trametinib, the MEK inhibitor, significantly inhibits ERK phosphorylation and PD-1/PD-L1 expression in malignant pleural mesothelioma [577]. Thus, 4-MU is a promising anti-cancer agent.

Other treatments such as normalization of ECM stiffness by photothermal depletion of cancer-associated fibroblasts in desmoplastic cholangiocarcinoma [578], and stiffness-based anti-angiogenic therapy in hepatoma [579] suggest that soften ECM stiffness is critical. Additionally, TNFα-CSG fusion protein, which interacts with laminin-nidogen complexes, decreases ECM stiffness and upregulates immuoinfiltration [580]. However, cholesterol depletion treatment could induce cancer stiffness and enhance T-cell immunotherapy [581]. Another research also demonstrates that softer ECM favors chemosensitivity in TNBC (via elevating NF-κB activity and compromising JNK activity) [582]. Thus, different strategies targeting ECM stiffness should be considered.

### Receptor surface domain blockage and drug delivery

Antibodies targeting ECM-associated receptors on the membrane of cancer cells can shut down the signal transduction from ECM to cancer cells, thus inhibit tumor progression. As previous introduced, RGDs are designed to recognize specific integrin and abrogates the function of integrin [175]. Besides, RGD-motif is an ideal guide to link anti-tumor agents, thus transport agents to target cells overexpressing specific integrins [583]. Moreover, RGDs can be used as developing agent such as a positron-emission tomography (PET) tracer to demonstrate the edge and volume of tumor, suggesting a potential role of ECM-associated monoclonal antibodies in oncogenic imaging [584, 585].

Similar to integrin, other receptors such as DDRs, CD44 [586] and syndecans are potential target for anti-cancer therapy. The ECD in DDRs interacts with collagen and stimulates activation of downstream signaling pathways. In DDR2, ECD domain suppress collagen I expression and mediates fibrillogenesis [587, 588]. Soluble DDR-ECD plays the same role [589]. Recently, a critical discovery unveils the role of DDR1-ECD in immunosuppression [484]. Precisely, ECD domain of DDR1 mediates collagen fibril alignment by interacting with collagen, then impede immune cell infiltration [484]. Other research indicates that methyl (CH3) and amino (NH2) mechanisms facilitated surface modification induce apoptosis in breast cancer [590]. Poly peptide of NH2-terminal fragment of zinc finger FYVE-type containing 21 (ZF21), which is the binding region of FAK, reduces metastasis progression [591]. These studies emphasize the strategy of domain blockage for impeding cell-ECM interaction.

For secreted proteins in ECM, blocking interaction domain of proteins to receptors by monoclonal antibody is an optimal selection. Fibrinogen-like globe (FBG) is a conserved domain in tenascins and mediates cytoskeletal re-organization by binding to fibronectin [592]. Monoclonal antibody targeting FBG can suppress tumor progression and enhance immunotherapy [508]. By the way, FBG domain on tenascin-X, a member of tenascins, supports its mechanic interaction with TGF-β, then induce maturation of TGF-β, highlights the potential role of FBG domain on other tenascins in TGF-β maturation [250, 593]. Additionally, extra-domain B (EDB) of fibronectin is an ideal target for drug delivery [594, 595]. A nanovehicle with a peptide bi-targeting EDB on fibronectin and FBG on TNC strikingly suppress tumor growth and increase survival of mice in vivo [596]. Thus, domains on ECM proteins are also optimal targets for drug delivery.

Though ECM are rich in proteins promoting tumor progression and immunosuppression in cancer, these proteins are indeed ideal targets for drug delivery. Among them, drug delivery system based on fibronectin has been concluded [597]. Nanoparticles featured by targeting ECM components are optimal to penetrate tumor tissue and deliver drugs. For instance, Collagenase IV modified nanoparticles loading doxorubicin can increase penetration of drug and with weaker side effects compared to nanoparticles without collagenase IV [598]. Overexpression of RHAMM, the specific receptor of HA, in cancer cells also provides a perfect target for HA nanogels [599]. Additionally, overexpression of ECM-associated enzymes in TME provides another strategy. For instance, a novel nanovehicle model with potential of hydrogen (pH)/ROS/MMP-2 triple-responsive will gradually release anti-cancer drug when meets MMP-2-rich, low pH and high density of intracellular ROS [600]. Then high concentration of intertumoral ROS induced by photothermal and acidic pH induces precisely delivery of sorafenib in vivo [600]. The similar strategy has been reported in a lipase-rich TME, which enhance the penetration of doxorubicin/monostearin constructed nanoparticles [601]. Importantly, to deal with the complex environment containing mechanic signals in ECM, novel nano materials automatically regulating mechanosensing according to ECM in cancer are on the way [602].

### CAR-T

Chimeric antigen receptor redirected T cells (CAR-T cells) are reengineered T cells from patients and shows significant therapeutic effects on cancers, especially on hematologic malignancies. Compared to monoclonal antibody, CAR-T shows the advantage of infiltration. For instance, human epidermal growth factor receptor 2 (Her-2) specific CAR-T shows a perfect penetration in tumor matrix better than Her-2 monoclonal antibodies [603]. However, the low therapeutic effects of CAT-T in solid tumor still suggests the emergency to enhance the ability of penetration of CAR-T. Thus, reengineered CAR-T cell with overexpression of heparinase, which can degrade ECM components and increases T-cell infiltration, strikingly inhibits tumor growth [604]. A therapeutic model with the combination of oncolytic adenovirus carried decorin with a CAR-T targeting carbonic anhydrase IX (CAIX) has been reported to induce ECM remodeling and immunoreaction in cancer recently [605]. Moreover, targeting stromal cells which are the primary cells generating ECM components, may be another selection. Fibroblast activation protein (FAD) is overexpressed in CAFs and associated with ECM remodeling in tumors and wound healing, and it is a potential target for ECM degradation and tumor imaging [606]. A study has demonstrated that FAP specific CAR-T significantly suppresses tumor progression and induce ECM degradation in PDAC [607]. Recently, an enhanced-affinity ligand for FAP has been developed and shows the potential for clinical application via CAR-T [608]. Additionally, CAR-147 modified macrophage increases T cell-infiltration by targeting MMPs in matrix [609]. Though less studies focus on the role of CAR-T in ECM remodeling, yet the model has the potential.

### Vitamin D

The role of activated Vitamin D (1,25-dihydroxy vitamin D3, or 1,25(OH)_2_ D_3_) in ECM remodeling has been reported that 1,25(OH)_2_ D_3_ stimulates generation of bone-associated proteins [610], as well as for OPN in bone [611] and TNC in mammary cells [612]. Most research targets the relationship between 1,25(OH)_2_ D_3_ and uterine leiomyomas. For instance, 1,25(OH)_2_ D_3_ level in plasma is negatively correlated with the risk of uterine leiomyomas [613]. TGF-β induces generation of fibronectin and collagen I whereas 1,25(OH)_2_ D_3_ overcomes the function in vitro [614, 615]. Long-term treatment of 1,25(OH)_2_ D_3_ shows the similar result in vivo [616]. It also inhibits the activation of Wnt4/β-catenin/mTOR pathway to suppress ECM generation [617], especially for mediator complex subunit 12 (MED12) mutation uterine leiomyomas [618]. Thus, 1,25(OH)_2_ D_3_ is a potential fibrosis inhibitor which may play a role in anti-malignant therapy.

Intriguingly, in vitro experiments suggest that 1,25(OH)_2_ D_3_ induce apoptosis in malignancy [619, 620] and differentiation [621] in malignancies. Moreover, generation of MMP-9 and MMP-13 is controlled by 1,25(OH)_2_ D_3_ in squamous cell carcinoma [622]. Recently, several studies focus on the function of 1,25(OH)_2_ D_3_ in ECM remodeling to suppress cancer progression. The interaction between PDAC cells and PSCs, and ECM remodeling induced by PSCs can be inhibited by treatment of the combination of Vitamin D receptor modulators and gemcitabine in vitro and in vivo [623]. Moreover, treatment of gemini-72, a vitamin D analog, normalizes ECM remodeling and induce apoptosis of prostatic precancerous cells in vivo [624]. However, the precise mechanism of Vitamin D-induced ECM remodeling in cancer is still unclear. To unveil the regulatory network may contribute to the ECM-based anti-cancer therapy.

## Future prospective and conclusion

Solid tumor is a high heterogeneity tissue composited by various cells and acellular components. This heterogeneity is not limited in the different composition of cancer cells and stromal cells, numerous ECM proteins with structural and/or bioactive role are critical to contribute to the formation of heterogeneity. Especially, multiple studies have indicated that the location, crosslink and modification of ECM proteins play an increasing role in ECM remodeling. To improve the therapeutic effects and outcomes of cancer patients, precise treatments targeting heterogeneity is the inevitable chosen. Nevertheless, almost all agents or cells selected to kill tumor cells are bound to face the resistance from ECM, the fenced wall around malignancy. The combination of anti-cancer therapy and anti-ECM treatment will be the next generation of ideal anti-malignancy strategy. This review has summarized the interaction between ECM and cancer, especially introduced the role of ECM in multiple hallmarks of cancer, including proliferation, anoikis, invasion, metastasis, angiogenesis, lymphangiogenesis, and immune escape. According to these mechanisms, we emphasize the importance of clinical applications targeting ECM to increase therapeutic effects and overcome drug resistance. ECM-targeting therapy has been proved to be effective in supporting anti-cancer treatment,and it is also a treasure which needs further exploration.

## Data Availability

Not applicable.

## Abbreviations

AMPK: AMP-activated protein kinase
ATP: Adenosine triphosphate
AHA1: ATPase homolog 1
ADAMTS3: A disintegrin and metalloproteinase with thrombospondin motifs 3
A2BR: Adenosine A2 receptor
apCAF: Antigen presenting cancer-associated fibroblast
ADAM8: A disintegrin and metalloproteinase domain 8
AMT: Aminomethylenethiophene
BCL: B-cell lymphoma
BRAF: B-Raf proto-oncogene
BAPN: Beta-aminopropionitrile
CTL: Cytotoxic T lymphocyte
C-P4H: Collagen prolyl 4-hydroxylase
CAF: Cancer-associated fibroblast
CD138/Sdc-1: Syndecan-1
CXCL3: C-X-C motif chemokine ligand 3
CCR2: C-X-C motif chemokine receptor 2
CSC: Cancer stem cell
COL10A1: Collagen type X alpha 1
CDK4/6: Cyclin-dependent kinases 4 and 6
CHI3L1/YKL-40: Chitinase-3-like protein 1
CCL2: C–C motif chemokine ligand 2
CCBE1: Collagen- and calcium-binding EGF domains 1
CSF1: Colony-stimulating factor 1
CAR-T: Chimeric antigen receptor redirected T cell
CAIX: Carbonic anhydrase IX
DDR2: Discoid protein domain receptor
DC: Dendritic cell
DAB2: Disabled homolog 2 mitogen-responsive phosphoprotein
ECM: Extracellular matrix
EC: Endothelial cell
ERK2: Extracellular signal-regulated kinase 2
EGFR: Epidermal growth factor receptor
ECT2: Epithelial cell transforming sequence 2
EMT: Epithelial-mesenchymal transition
EPHA2: Ephrin type-A receptor 2
EMILIN2: Elastin microfibrillar interface protein 2
ECD: Extracellular domain
EDB: Extra-domain B
FGF: Fibroblast growth factor
FAK: Focal adhesion kinase
FOXO1: Forkhead box protein O1
FBG: Fibrinogen-like globe
FAD: Fibroblast activation protein
GTPase: Guanosine triphosphatase
G3BP2: Ras-GTPase activating protein SH3 domain-binding protein 2
GLUT-1: Glucose transporter protein type 1
GEF: Guanine nucleotide-exchange factor
GATA2: GATA binding protein 2
GFAT1: Glutamine-fructose amidotransferase 1
HIF-1: Hypoxia-inducible factor 1
HSPG: Heparan sulfate proteoglycan
HA: Hyaluronan/hyaluronic acid
HAS2: Hyaluronan synthase 2
HSC: Hepatic stellate cell
HSP: Heat shock protein
hsPxd01: Heme peroxidase human peroxidasin 1
HAPLN1: Hyaluronan and proteoglycan link protein 1
Her-2: Human epidermal growth factor receptor 2
IL-8: Interleukin-8
IGFBP2: Insulin-like growth factor binding protein 2
iCAF: Inflammatory cancer-associated fibroblast
IFN-γ: Interferon gamma
JNK: C-Jun N-terminal kinase
KLF5: Kruppel-like factor 5
LOX: Lysyl oxidase
LOXL: Lysyl oxidase-like proteins
LN-5γ2: Laminin-5γ2
LAIR: Leukocyte-associated Ig-like receptor
LEC: Lymphatic endothelial cell
LIF: Leukemia inhibitory factor
MMP: Matrix metalloproteinase
MAPK: Mitogen-activated protein kinase
MDA-9: Melanoma differentiation associated gene-9
mTOR: Mechanistic target of rapamycin
MSC: Mesenchymal stromal cell
MMRN2: Multimerin-2
myCAF: Cancer-associated myofibroblasts
MDSC: Myeloid-derived suppressor cell
MED12: Mediator complex subunit 12
NK: Natural killer
NF-κB: Nuclear factor-kappaB
NSCLC: Non-small cell lung cancer
NADPH: Nicotinamide adenine dinucleotide phosphate
NRF2: Nuclear factor E2-related factor
N-WASP: Neural Wiskott-Aldrich syndrome protein
NMIIAH: Non-muscle myosin IIA
NO: Nitric oxide
OPN/SPP-1: Osteopotin
OXPHOS: Oxidative phosphorylation
PDAC: Pancreatic ductal adenocarcinoma
PI3K: Phosphoinositide 3-kinase
PDO: Patient-derived organoid
PSC: Pancreatic stellate cell
PRP4K: Pre-mRNA splicing factor 4 kinase
PDGFB: Platelet-derived growth factor-BB
PTPRK: Receptor-type tyrosine-protein phosphatases kappa
PFKFB3: Phosphofructokinase-2/fructose-2, 6-bisphosphatase 3
PPP: Pentose phosphate pathway
Prrx1: Paired related homeobox 1
PLOD2: Procollagen-lysine, 2-oxoglutarate 5-dioxygenase 2
PD-L1: Programmed death ligand 1
PD-1: Programmed death 1
ROS: Reactive oxygen species
RHAMM/CD168: Receptor for hyaluronan-mediated motility
ROCK: Rho-associated coiled-coil containing kinase
RGD: Arg-Gly-Asp
RREB1: RAS-responsive element binding protein 1
PC: Pyruvate carboxylase
RIPK-1: Receptor-interacting protein kinase 1
SPARC: Secreted proteins acidic and rich in cysteine/osteonectin
SKP2: S-phase kinase associated protein 2
STAT3: Signal transducer and activator of transcription 3
SNAIL: Snail family transcriptional repressor
Setd2: SET domain containing 2
SOX4: SRY-Box transcription factor 4
SMAD4: Small mothers against decapentaplegic protein 4
SOD2: Superoxide dismutase 2
SHP-1: Src homology 2 domain-containing protein tyrosine phosphatase 1
SMA: Spinal muscular atrophy
TME: Tumor microenvironment
THBS/TSP: Thrombospondin
Treg: Regulatory T cell
TAM: Tumor-associated macrophage
TRC: Tumor-repopulating cell
TLR-4: Toll-like receptor 4
TNC: Tenascin-C
TAZ/WWTR1: WW domain containing transcription regulator
TGF-β-2: Transforming growth factor β2
Tet2: Ten-eleven translocation 2
TWIST: Twist family basic helix-loop-helix transcription factor
TRIO: Trio Rho guanine nucleotide exchange factor
TGFBI/βig-H3: Transforming growth factor beta-induced protein
TRPM7: Transient receptor potential melastatin 7
TXNIP: Thioredoxin-interacting protein
TNBC: Triple negative breast cancer
TIMP2: Tissue inhibitors of metalloproteinase 2
TNFα: Tumor necrosis factor alpha
UBTD1: Ubiquitin domain containing 1
UDP: Uridine diphosphate
VEGF: Vascular endothelial growth factor
Vitamin D: 1,25-dihydroxy vitamin D3
WISP: WNT1-inducible signaling pathway protein
WDR5: WD repeat domain 5
YAP: Yes1 associated transcriptional regulator
ZFP36: Zinc finger protein 36
ZEB1: Zinc finger E-box binding homeobox 1
ZF21: Zinc finger FYVE-type containing 21
4-MU: 4-methylumbelliferone
